# Iridium oxide nanoribbons with metastable monoclinic phase for highly efficient electrocatalytic oxygen evolution

**DOI:** 10.1038/s41467-023-36833-1

**Published:** 2023-03-04

**Authors:** Fan Liao, Kui Yin, Yujin Ji, Wenxiang Zhu, Zhenglong Fan, Youyong Li, Jun Zhong, Mingwang Shao, Zhenhui Kang, Qi Shao

**Affiliations:** 1 Institute of Functional Nano & Soft Materials (FUNSOM), Jiangsu Key Laboratory for Carbon-Based Functional Materials & Devices, Soochow University, 199 Ren’ai Road, Suzhou, 215123 Jiangsu China; 2 College of Chemistry, Chemical Engineering and Materials Science, Soochow University, Suzhou, Jiangsu 215123 China; 3 Macao Institute of Materials Science and Engineering, Macau University of Science and Technology, Taipa, 999078 Macau, SAR China

**Keywords:** Metamaterials, Nanowires, Electrocatalysis, Electrocatalysis, Nanowires

## Abstract

Metastable metal oxides with ribbon morphologies have promising applications for energy conversion catalysis, however they are largely restricted by their limited synthesis methods. In this study, a monoclinic phase iridium oxide nanoribbon with a space group of C2/m is successfully obtained, which is distinct from rutile iridium oxide with a stable tetragonal phase (P42/mnm). A molten-alkali mechanochemical method provides a unique strategy for achieving this layered nanoribbon structure via a conversion from a monoclinic phase K_0.25_IrO_2_ (I2/m (12)) precursor. The formation mechanism of IrO_2_ nanoribbon is clearly revealed, with its further conversion to IrO_2_ nanosheet with a trigonal phase. When applied as an electrocatalyst for the oxygen evolution reaction in acidic condition, the intrinsic catalytic activity of IrO_2_ nanoribbon is higher than that of tetragonal phase IrO_2_ due to the low *d* band centre of Ir in this special monoclinic phase structure, as confirmed by density functional theory calculations.

## Abstract (web summary)

Well-defined metastable phase nanostructures are a core issue for catalyst design. Here, the authors report metastable monoclinic phase IrO2 nanoribbons obtained via a molten-alkali mechanochemical method, which exhibit intrinsic high performance towards the acidic oxygen evolution reaction.

## Introduction

Electrolyzing water into clean and renewable hydrogen in acidic electrolytes is an effective technique to relieve energy and environmental protection concerns^1–4^. An efficient electrocatalyst for an anodic oxygen evolution reaction (OER) significantly improves the energy conversion efficiency because most of the energy consumption of electrolytic water splitting occurs at the anode^5–7^. Iridium oxide (IrO_2_) is considered to be a state-of-the-art catalyst for OER that can withstand harsh acidic condition^8–11^. However, the previously reported IrO_2_ catalysts lack high intrinsic activity against the slow kinetics of OER.

Constructing a metastable nanostructure is a promising method to pursue superior catalytic properties^12–17^. Naturally occurring thermodynamically stable IrO_2_ is commonly found in a rutile phase with pristine regular [Ir-O_6_] units linked by shared edge and corner modes. Earlier works have demonstrated that the intrinsic activity of IrO_2_ is closely associated with [Ir-O_6_] unit linkage construction^18–21^ and lattice distortion in [Ir-O_6_] octahedrons^22–24^. Therefore, designing a metastable nanostructure with different unit linkages may provide a completely distinct active surface for electrocatalysis and a deep understanding of the structure-activity relationship^25–29^. Low-dimensional materials with increased surface energies provide substrates for the development of advanced catalysts due to their inherent anisotropic properties, quantum confinement characteristics and edge effects^30,31^. However, IrO_2_ with an unconventional crystal phase and nanoribbon morphology has not yet been reported.

Motivated by all these possibilities, in this work, a metal oxide comprising monoclinic phase layered IrO_2_ nanoribbons (IrO_2_NRs), with the space group C2/m (12), is prepared. The Ir-O coordination polyhedrons in the IrO_2_NRs maintain an octahedral configuration, while the connections of the octahedral subunits are in an edge-sharing mode. The space group of the IrO_2_NR is C2/m (12), which is totally different from that of Rutile IrO_2_ (P4_2_/mnm (136)). A molten-alkali mechanochemical method promotes metastable phase formation from a precursor of monoclinic K_0.25_IrO_2_ (I2/m (12)). The formation mechanism of the IrO_2_NR and the further conversion of IrO_2_ nanosheets (IrO_2_NSs) are clearly determined according to an experimental observation. Due to the specific phase construction and nanoribbon morphology, the IrO_2_NR exhibits superior OER activity and stability in acidic electrolytes. Theoretical calculations have been carried out to explain the high intrinsic activity of IrO_2_NR.

## Results

### Morphology and structure of IrO_2_NRs

IrO_2_NRs are fabricated via a molten-alkali mechanochemical method in a homemade mechano-thermal reactor, which provides unique synthetic conditions, such as high temperature, a strong alkaline media and a continuous grinding force. The fabrication process is schematically shown in Supplementary Fig. 1. The raw materials (IrCl_3_ and KOH) are first stirred continuously at 150 °C in a Teflon mortar. Then, a dark blue slurry forms and is transferred to the homemade mechano-thermal reactor, where it is heated at 700 °C for 2 h to generate IrO_2_NRs.

**Fig. 1:**
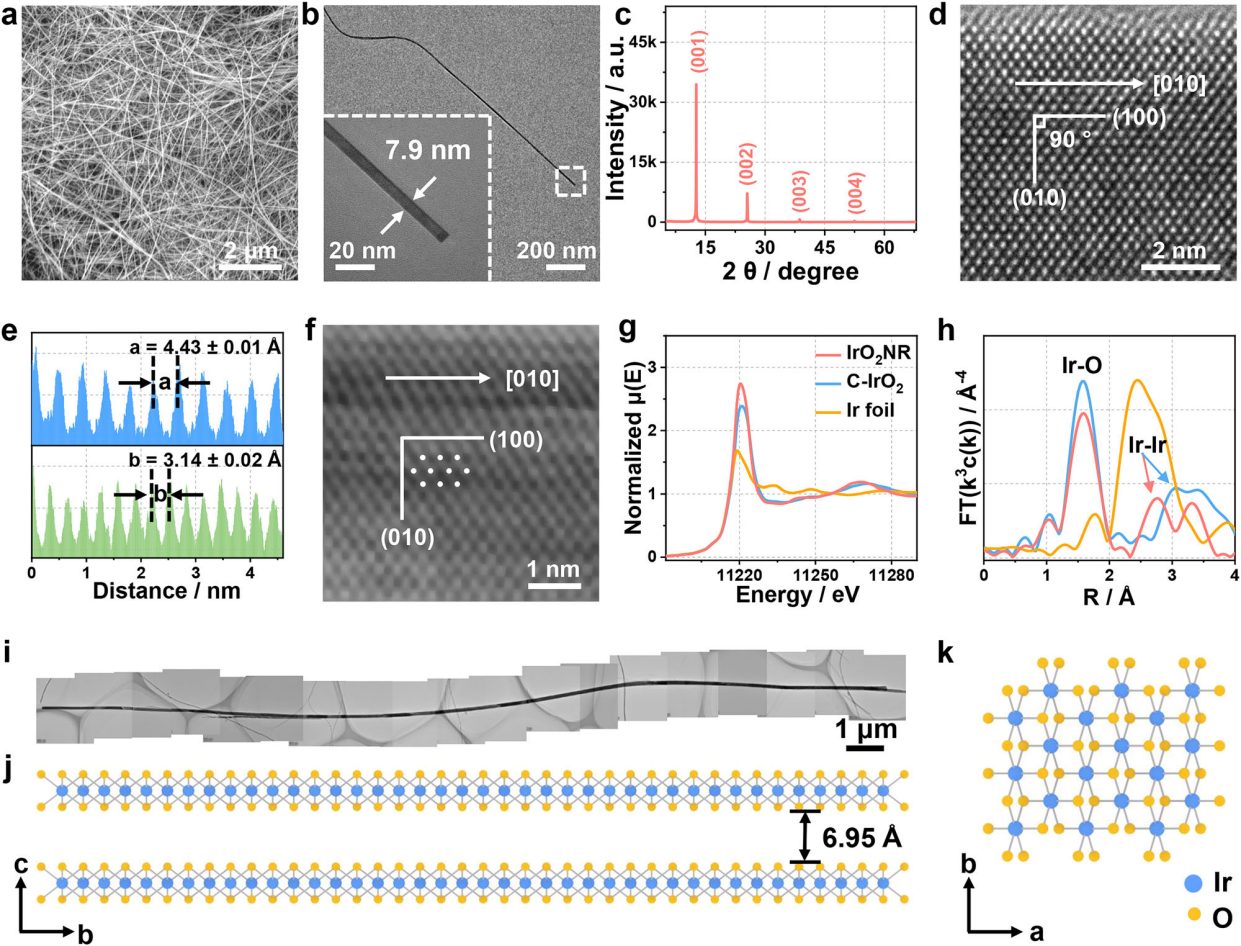
Morphology and structure of the IrO_2_NR. **a** SEM image showing the uniform distribution of the IrO_2_NR. **b** TEM image of a single IrO_2_NR. The insert is the enlargement of the tail end of the ribbon with a width of 7.9 mm. **c** XRD pattern. **d** HRTEM image. **e** Line scan of the HRTEM image indicated by the (100) and (010) planes. **f** HAADF–STEM image for the IrO_2_NR. **g** XANES spectra and **h** FT–EXAFS spectra for the IrO_2_NR, C-IrO_2_ and Ir foil. **i** A typical long IrO_2_NR with a length of 22.53 μm. **j**, **k** Pictorial illustration of the crystal structure for the IrO_2_NR from the (**j**) ***a*** direction and (**k**) ***c*** direction.

The morphologies of the IrO_2_NRs on a large scale are first observed by scanning electron microscopy (SEM) as shown in Fig. 1a. All the samples exhibit a uniform nanoribbon structure. The dense and flexible IrO_2_NRs are entangled. The transmission electron microscopy (TEM) image shown in Supplementary Fig. 2 displays a nanoribbon morphology at a high magnification. A typical TEM image of a single IrO_2_NR is shown in Fig. 1b. From the enlarged figure of the tail end, the width of this nanoribbon is 7.9 nm. The average width is 10.0 nm, based on 500 individual samples (Supplementary Fig. 3). Pictures of several distinctive IrO_2_NRs are displayed in Supplementary Fig. 4 to show their widths and lengths. A typical long IrO_2_NR is shown in Supplementary Fig. 4a, with a length of ~22.53 μm. A thin IrO_2_NR with a width of 4.9 nm is shown in Supplementary Fig. 4b. The lattice tension/compression can be observed at the edges of the IrO_2_NRs according to the HRTEM images (Supplementary Fig. 5). The average lattice strain is calculated by the Williamson-Hall equation based on the XRD data (Supplementary Fig. 6), which is about 0.388%. Although the lattice strain exists in IrO_2_NR, the effect on activity may be limited due to its low value when we compared with the stain in previous reference about IrO_2_ for OER catalysis^32^. The thicknesses of the IrO_2_NRs range from ~6.0 to 12.0 nm, as confirmed by atomic force microscopy (AFM) images (Supplementary Fig. 7). The resistivity of the IrO_2_NR is estimated to be 1.2 × 10^–6^ Ω m, indicating its high conductivity behaviour (Supplementary Fig. 8).

Notably, an X-ray powder diffractometer (XRD) pattern of IrO_2_NR (Fig. 1c) exhibits four peaks with proportional *d* values, indicating that IrO_2_NR has a layered structure with a *c*-axis spacing of 6.95 ± 0.03 Å. The EDS spectrum shown in Supplementary Fig. 9a indicates that the IrO_2_NRs are composed of only Ir and O, with atomic ratios close to 1: 2. A high-angle annular dark-field scanning transmission electron microscopy (HAADF–STEM) image and a corresponding elemental mapping (Supplementary Fig. 9b–d) reveal the homogeneous distributions of Ir and O in the IrO_2_NRs.

The high-resolution transmission electron microscopy (HRTEM) image shown in Fig. 1d displays the well-resolved atomic structure of the IrO_2_NR. The lattice fringe spacing and the crystal plane angle are measured accordingly. Based on the selection principle of the Bravais lattices, two directions, defined as *a* and *b* with crystal plane angles of 90°, are selected to represent the unit cell. The parameters of the unit cell are as follows: *a* = 4.43 ± 0.01 Å, *b* = 3.14 ± 0.02 Å, and *γ* = 90° (Fig. 1d, e). The aberration-corrected HAADF–STEM image in Fig. 1f shows the atomic arrangement of Ir, which is in accordance with that in the HRTEM image. The cell parameters of the IrO_2_NRs are obviously different from those of Rutile IrO_2_ (Supplementary Table 1)^33^. The selective-area electron diffraction (SAED) pattern in Supplementary Fig. 10 indicates the crystalline nature of the IrO_2_NRs. The IrO_2_NRs show layered structures and tend to lay flat on the copper mesh, making it difficult to find a plane that is perpendicular to the [001] direction. Then, the IrO_2_NRs are embedded in epoxy resin and sliced into pieces, which are picked up with copper mesh. From the HRTEM image of a cross section of IrO_2_NR (Supplementary Fig. 11), a distance of 0.69 nm that belongs to (001) plane is observed, which is in accordance with the XRD results. Additionally, due to the preferred orientation of the IrO_2_NR, no peaks other than the layered structure peaks are observed in the XRD pattern.

The structure and oxidation states of the IrO_2_NR are analysed by synchrotron X-ray absorption spectroscopy (XAS) using commercial IrO_2_ (C-IrO_2_, rutile phase) and Ir foil as the reference. An Ir-L_3_ edge X-ray absorption near-edge spectroscopy (XANES) image of the IrO_2_NR is shown in Fig. 1g. The absorption edge and spectral shape of the IrO_2_NR are similar to those of C-IrO_2_, indicating an oxidation state of +4 for Ir in the IrO_2_NR^34^. Fourier transformed extended X-ray absorption fine structure spectra (FT–EXAFS) in Fig. 1h show the scattering profile as a function of the radial distance from the central absorbing Ir atom. The spectra here are plotted without phase correction. For the IrO_2_NR, the main peak is located at ~1.6 Å, corresponding to the Ir-O distance in the [Ir-O_6_] octahedron, which is similar to the first coordination shell of Rutile IrO_2_^35^. Additionally, the second Ir-Ir shell is clearly observable in the IrO_2_NR, which is located at ~2.76 Å; this location is lower than that in C-IrO_2_, indicating that the interconnection of the [Ir-O_6_] octahedron follows another model in the IrO_2_NR^36^. According to the HRTEM image and the XAS data, the structure of the IrO_2_NR (Fig. 1i) from the *a* and *c* directions is determined and shown in Fig. 1j, k. There is a two-fold rotation axis through the *b* direction with a vertical reflection. The symmetry space group of the IrO_2_NR is determined to be monoclinic C2/m (12), and the corresponding crystallographic information is shown in Supplementary Table 1. All the above results conclude that a layered IrO_2_ nanostructure with monoclinic C2/m (12) phase is successfully obtained, the determination of which is schematically shown in Supplementary Fig. 12. A simulated XRD pattern based on the structure of the IrO_2_NR is shown in Supplementary Fig. 13. As seen from the figure, peaks at ~12 and 25° are observed. The disappearance of other peaks is due to the preferred orientation of the IrO_2_NR. The exposed crystal faces obtained from the [001] direction in the SAED patterns (Supplementary Fig. 10) are in accordance with the stimulated XRD pattern of the IrO_2_NR structure, confirming its novel structure.

### Formation mechanism of IrO_2_NRs

Considering the unique phase and dimensionality property of IrO_2_NRs, their formation processes are investigated in detail. The initial slurry obtained by heating the mixture of ethanol solution of IrCl_3_ and KOH at 150 °C is taken out directly and characterized by XRD and TEM as shown in Supplementary Fig. 14. The XRD pattern of the slurry sample before washing is shown in Supplementary Fig. 14a. Although the initial slurry is very complex due to the strong alkaline medium and hydrate formation, the main peaks can be indexed as KIrO_3_, KOH, KO_1.58_H_0.42_ and KOH·H_2_O. The slurry sample shows a droplet-like morphology as seen in the TEM image in Supplementary Fig. 14b. The slurry at this stage is easily dissolved in water during the washing process. After washing, there is no solid product. The XRD patterns and morphology images of the products obtained at reaction temperatures from 300 to 600 °C are shown in Supplementary Fig. 15. When the reaction temperature ranges from 300 to 400 °C, the samples are amorphous and show no unique shapes (Supplementary Fig. 15a–f). Upon increasing the temperature to 500 °C (Supplementary Fig. 15g–i), the bulk nanoparticles become fluffy. The XRD pattern of the sample obtained at 500 °C shows monoclinic structure K_0.25_IrO_2_ (PDF No. 85-2185)^37^. The chemical composition of K_0.25_IrO_2_ obtained at a reaction temperature of 500 °C is further investigated as shown in Supplementary Fig. 16. The HRTEM image (Supplementary Fig. 16a) shows that the crystal plane of (211) of K_0.25_IrO_2_. The SAED pattern shows that K_0.25_IrO_2_ is polycrystalline (Supplementary Fig. 16b). The bright ring corresponds to the (211) plane (JCPDS No. 85-2185). The corresponding elemental mapping results indicate the existence of K, Ir and O elements (Supplementary Fig. 16c–f). When the temperature reaches 600 °C (Supplementary Fig. 15j–l), small nanoribbon structures can be found in the TEM images. The characteristic XRD peaks of K_0.25_IrO_2_ begin to fade, and the layered peaks gradually form and become apparent. From these data, we speculate that the IrO_2_NR originates from K_0.25_IrO_2_ and transforms into IrO_2_NR with increasing temperature.

More comparison experiments are performed to further indicate the important roles of alkaline conditions. The K_0.25_IrO_2_ sample (obtained at the reaction temperature of 500 °C) is washed with water to remove the KOH and then calcined at 700 °C for 2 h. The XRD pattern shows that it transforms into Rutile IrO_2_ (Supplementary Fig. 17a), the morphology of which is a nonuniformly shaped nanoparticle, as shown in the TEM image in Supplementary Fig. 17b. Then, KOH is added to K_0.25_IrO_2_, and the fabrication process of the IrO_2_NR calcined at 700 °C for 2 h is repeated. The XRD pattern of the obtained sample shows layered peaks (Supplementary Fig. 17c). The TEM image indicates that a nanoribbon morphology can form during this process (Supplementary Fig. 17d), which confirms that KOH is very important for the formation of IrO_2_NR.

Next, the chemical states of Ir and O in the IrO_2_NR and K_0.25_IrO_2_ are investigated by X-ray photoelectron spectroscopy (XPS). XPS measurements are conducted for C-IrO_2_ and C-Ir/C for comparison. As shown in Supplementary Fig. 18a, K is present in K_0.25_IrO_2_. However, only Ir and O are detected in IrO_2_NR and C-IrO_2_, indicating the purity of our samples. Then, the Ir 4 *f* fine spectra of the four samples are deconvoluted (Supplementary Fig. 18b). For C-IrO_2_, Ir 4*f*_7/2_ at 62.2 eV and Ir 4*f*_5/2_ at 65.2 eV correspond to the reported Rutile IrO_2_, with two accompanying satellite peaks^38,39^. The oxidation states of Ir in the IrO_2_NRs are close to C-IrO_2_, which is 0.3 eV higher than that of K_0.25_IrO_2_. No metallic Ir is detected in the IrO_2_ samples, as determined by the binding energy of Ir in C-Ir/C^40^. The O 1 *s* spectrum of IrO_2_NR can be deconvoluted into three peaks, including lattice oxygen (O_Ir–O_), unsaturated oxygen (O_V_) and adsorbed oxygen $({{{{{{\rm{O}}}}}}}_{{{{{{{\rm{H}}}}}}}_{2}{{{{{\rm{O}}}}}}})$ (Supplementary Fig. 18c).

The XANES and EXAFS spectra of the IrO_2_NRs are compared to those of K_0.25_IrO_2_. The oxidation state of Ir in K_0.25_IrO_2_ is slightly lower than that in the IrO_2_NR (Supplementary Fig. 19a), as seen from the first derivative of the Ir-L_3_ edge curves between the IrO_2_NR and K_0.25_IrO_2_ samples (Supplementary Fig. 19b). The second Ir-Ir shell of K_0.25_IrO_2_ in the FT–EXAFS spectrum (Supplementary Fig. 19c) occurs due to the edge shared [Ir-O_6_] octahedron, the location of which is close to that of the IrO_2_NR (Supplementary Fig. 19c,d)^41^. Combined with the K_0.25_IrO_2_ detected during the fabrication process, we conclude that the structure of the IrO_2_NR evolves from that of K_0.25_IrO_2_ by inheriting the edge sharing [Ir-O_6_] octahedron linkage mode.

Another interesting phenomenon is that when we further increase the reaction temperature to 800 °C for 2 h, most of the IrO_2_NR transforms into a nanosheet morphology, which is defined as IrO_2_NS (Supplementary Fig. 20). The XRD pattern of the IrO_2_NS (Supplementary Fig. 20a) is similar to that of the IrO_2_NR; however, that the peak intensity of IrO_2_NS is larger than that of IrO_2_NR, indicating that the crystallinity of IrO_2_NS is higher than that of IrO_2_NR, which may be due to the high reaction temperature. The SEM and TEM images in Supplementary Fig. 20b–d clearly show that most samples exhibit nanosheet morphologies with several nanoribbons. The HRTEM and SAED images (Supplementary Fig. 20e, f) show that different crystal structures between the IrO_2_NS and IrO_2_NR. The IrO_2_NS can be assigned to trigonal phase with a space group of P-3m1 (164)^42^. The structural parameters of the IrO_2_NSs are listed in Supplementary Table 1.

**Fig. 2:**
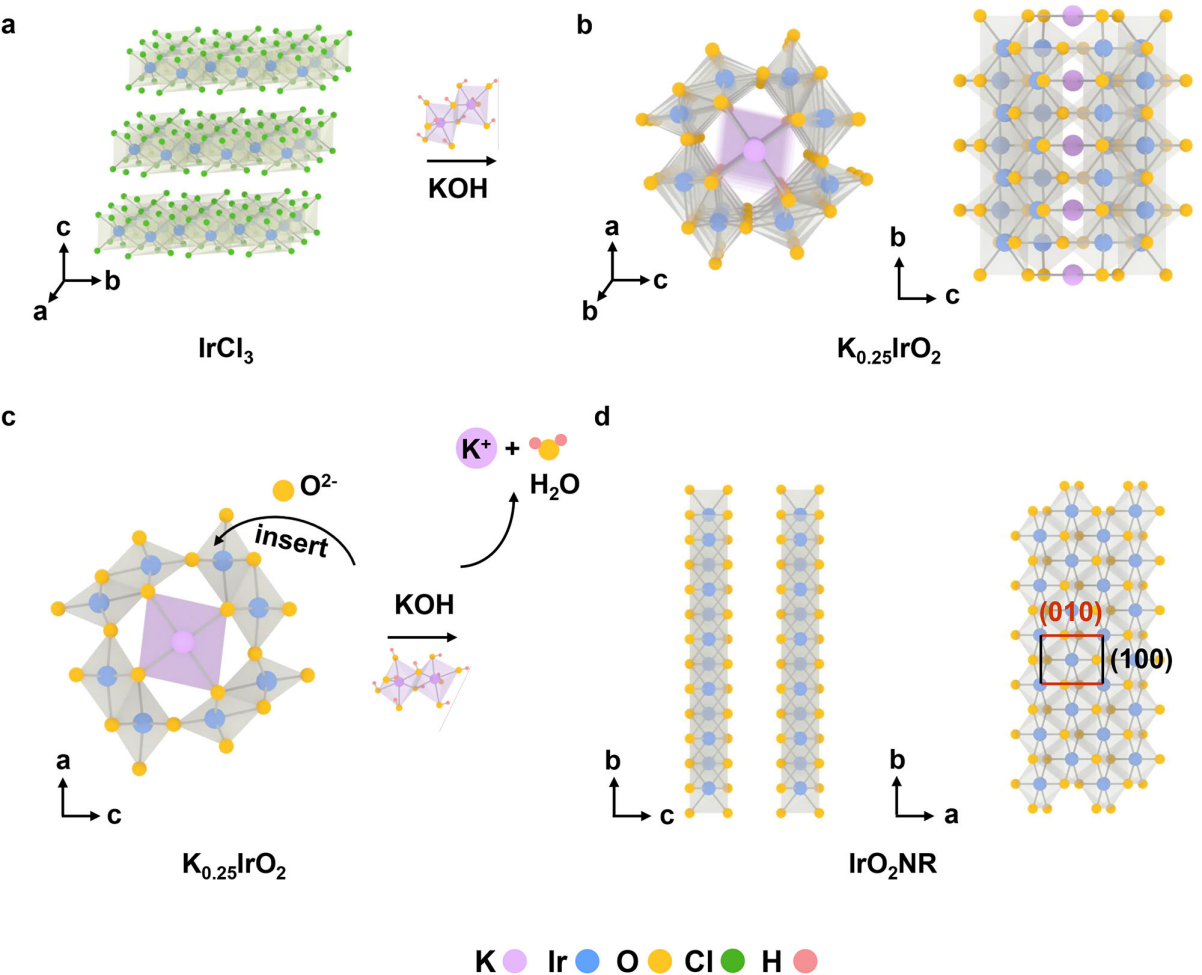
Structural evolution of the IrO_2_NR. **a**, **b** From the raw materials of IrCl_3_ and KOH to K_0.25_IrO_2_. **c**, **d** From K_0.25_IrO_2_ to the IrO_2_NR. Purple, blue, yellow, green and pink balls represent K, Ir, O, Cl and H elements, respectively.

From the above results, the structural transformation process of this unique IrO_2_NR is proposed in Fig. 2. First, IrCl_3_ reacts with KOH by the molten-alkali mechanochemical method to form K_0.25_IrO_2_ (Fig. 2a, b). Then, the O^2–^ ions, originating from KOH at high temperature, attack the corner-connected octahedron of K_0.25_IrO_2_ (Fig. 2c) to form the IrO_2_NR structure (Fig. 2d). During fabrication at different temperatures, the IrO_2_NR maintains the same [Ir-O_6_] edge linkage mode as K_0.25_IrO_2_^43^. The growth direction of the IrO_2_NR is defined according to the crystal-axis orientation as K_0.25_IrO_2_, which means that the IrO_2_NR is oriented in the *b* direction. Therefore, the (010) and (100) crystal planes are identified as marked in Fig. 2d. After the oxygen bonds break in K_0.25_IrO_2_, the [Ir-O_6_] subunits connect through an edge-edge mode (Supplementary Fig. 21a); the number of columns of [Ir-O_6_] subunits that perpendicular to the *b* direction must be even, as demonstrated by Supplementary Fig. 21b, c. The IrO_2_NR retains the nanoribbon morphology in the final product because of the addition of ethanol to the raw materials. Ethanol adsorbs on the products during the fabrication process and transfers to amorphous carbon at high temperature, which slows the speed of the ribbon-to-ribbon connection. When the reaction temperature further increases to 800 °C, the edges of the IrO_2_NR connect and form a nanosheet morphology with a slightly changed lattice constant, as shown in Supplementary Fig. 20g.

### Electrocatalytic activity of the IrO_2_NR for OER

**Fig. 3:**
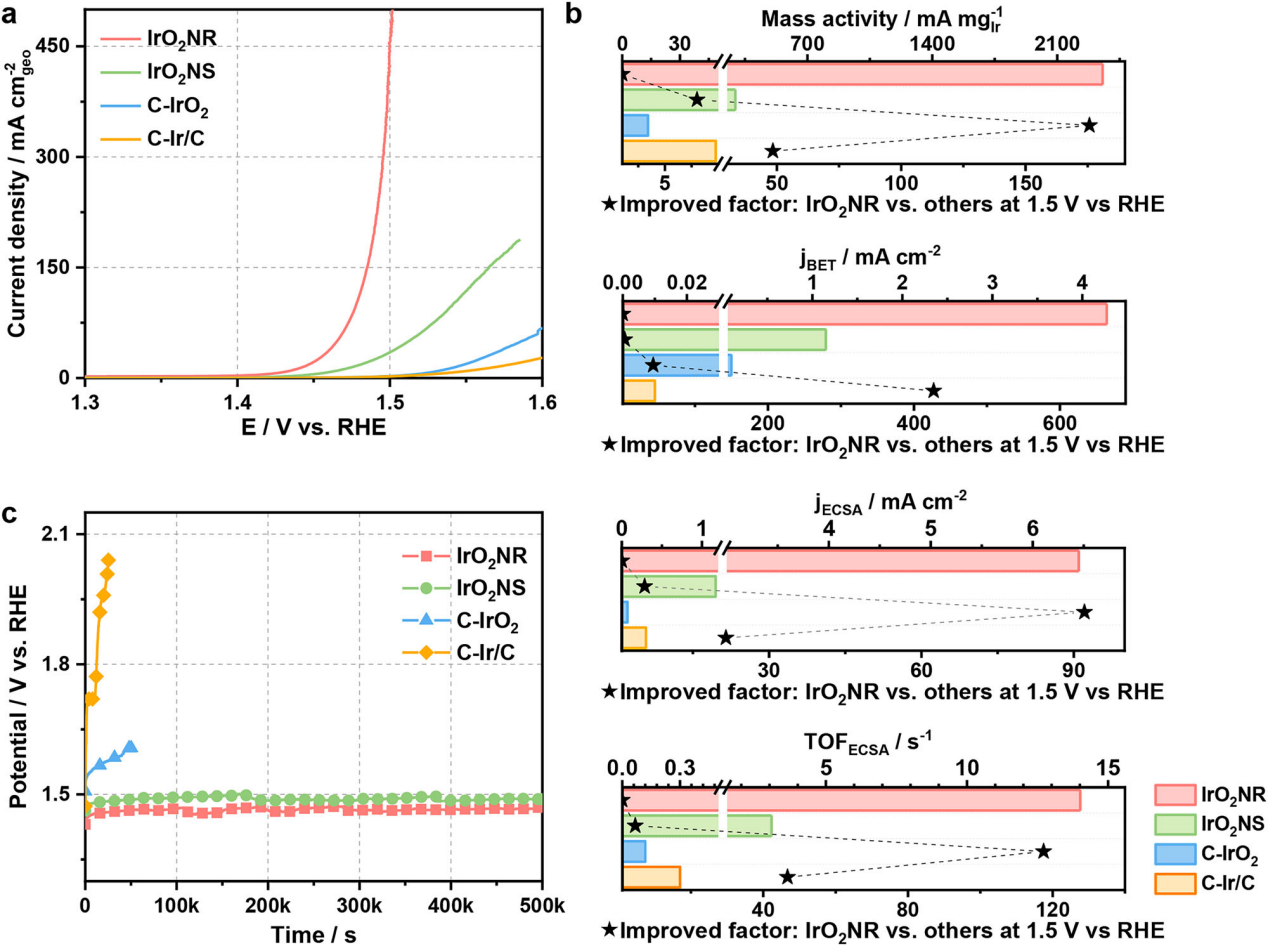
The OER activities of the IrO_2_NR, IrO_2_NS, C-IrO_2_ and C-Ir/C in 0.5 M H_2_SO_4_. **a** LSV curves of the IrO_2_NR, IrO_2_NS, C-IrO_2_ and C-Ir/C samples. **b** Mass activities and specific activities based on the BET areas and ECSA areas determined by the mercury underpotential deposition method and TOF values based on the ECSA values at potential of 1.5 V vs. RHE. **c** Chronopotentiometric curves of the IrO_2_NR, IrO_2_NS, C-IrO_2_ and C-Ir/C samples at a constant current density of 10 mA cm^–2^.

The OER activity of the IrO_2_NR is evaluated in an O_2_-saturated 0.5 M H_2_SO_4_ electrolyte using a standard three-electrode system. The Hg/HgCl_2_ reference electrode (saturated calomel electrode; SCE) is calibrated before the OER test (Supplementary Fig. 22)^44^. C-IrO_2_ and C-Ir/C are tested as benchmarks. The XRD patterns of C-IrO_2_ and C-Ir/C are shown in Supplementary Fig. 23, which indicate that C-IrO_2_ has a rutile structure, and that Ir in Ir/C has a face-centred cubic structure. Samples obtained at different steps, including K_0.25_IrO_2_ and IrO_2_NS, are also employed for comparison. A full connection between the conductive substrate (glassy carbon electrode), IrO_2_NR and electrolyte is required to perform electrochemical measurements. To verify the connection phenomena, a contact angle experiment is conducted. IrO_2_NR and C-IrO_2_ are pressed into tablets. Then, the contact angle of each tablet is tested (Supplementary Fig. 24). Interestingly, the H_2_SO_4_ (0.5 M) droplet can permeate the IrO_2_NR (Supplementary Fig. 24a, c) faster than it can permeate C-IrO_2_ (Supplementary Fig. 24b, d), which may be because the IrO_2_NR can form a network structure and exhibit good electrical contact between the conductive substrate and the electrolyte. Linear sweep voltammetry (LSV) curves are obtained at a scan rate of 5 mV s^–1^ with 95% *i*R-compensation (Fig. 3a). The IrO_2_NR exhibits excellent OER activity with low overpotential of 205 mV to achieve a current density of 10 mA cm^–2^, which is 98 and 117 mV lower than those of C-IrO_2_ and C-Ir/C, respectively. K_0.25_IrO_2_ is a poor OER catalyst with an overpotential of 344 mV at 10 mA cm^–2^ (Supplementary Fig. 25). The OER performance of the IrO_2_NS is compared to that of IrO_2_NR. IrO_2_NS exhibits a high OER activity, with an overpotential of 235 mV@10 mA cm^–2^, which is only 30 mV lower than that of IrO_2_NR. To comprehensively analyse the catalytic kinetics of OER, the Tafel slopes of the four catalysts are calculated, and they are displayed in Supplementary Fig. 26. The Tafel slope of the IrO_2_NR is 46.2 mV dec^–1^, which is smaller than those of the IrO_2_NS (54.2 mV dec^–1^), C-IrO_2_ (61.2 mV dec^–1^), and C-Ir/C (97.3 mV dec^–1^), indicating the fast OER kinetics for the IrO_2_NR.

A comparison of the overpotential and Tafel slope indicates that IrO_2_NRs are more beneficial for OER catalysis. The performance of K_0.25_IrO_2_ is too poor for further comparison. To explore the origin of the high activity level, the specific surface areas and electrochemically active surface areas (ECSAs) are analysed by a Brunauer–Emmett–Teller (BET) method and a mercury underpotential deposition method, respectively^45^. The results are shown in Supplementary Figs. 27 and 28, and the data are listed in Supplementary Table 2. The BET area of the IrO_2_NR is 47.3 m^2^ g^–1^, which is larger than those of the other IrO_2_ catalysts. The BET area of C-Ir/C is much larger than that of the IrO_2_ catalysts due to the addition of carbon black. The ECSAs are calculated to show the catalytic active sites of the catalysts, where the ECSA of the IrO_2_NR is 31.3 m^2^ g^–1^, which is larger than those of C-IrO_2_ (15.5 m^2^ g^–1^) and the IrO_2_NS (21.6 m^2^ g^–1^).

The current densities at 1.5 V vs. RHE based on different standards are calculated and shown in Fig. 3b. The detailed data are listed in Supplementary Table 3. The mass activity, specific activity and turnover frequency (TOF) values of the IrO_2_NR are obviously larger than those of C-IrO_2_ and C-Ir/C. The mass activity of the IrO_2_NR at 1.5 V vs. RHE is 2354.5 mA mg_Ir_^–1^, which is 175.7 times larger than that of C-IrO_2_ (13.4 mA mg_Ir_^–1^). The specific activities based on the BET and ECSA of the IrO_2_NR are 42.7 and 92.1 times larger than those of C-IrO_2_. Notably, the performance of the IrO_2_NR is better than that of the IrO_2_NS. The mass activity of the IrO_2_NR is 8.0 times larger than that of the IrO_2_NS (295.7 mA mg_Ir_^–1^). The specific activities based on the BET and ECSA of the IrO_2_NR are also 3.7 and 5.5 times larger than those of the IrO_2_NS. On the basis of ECSA, a high TOF of 14.01 s^–1^ is achieved by the IrO_2_NR at 1.5 V vs. RHE, indicating the high quality of its active sites. The OER performance of the IrO_2_NR is compared to those of other Ir-based catalysts, as shown in Supplementary Table 4. The specific activity levels of the IrO_2_NR normalized to the BET and ECSA areas are compared (Supplementary Table 5), demonstrating the excellent OER activity in Ir-based catalysts. The Faradic efficiency (FE) is a critical indicator that shows the reaction route. The produced oxygen of IrO_2_NR at a current density of 20 mA cm^–2^ is collected at 30-min intervals (Supplementary Fig. 29). The FEs of IrO_2_NR in the 120 min test are maintained above 95.0%, which suggests that the observed catalytic current originates from the water oxidation process.

In addition to activity, stability is an important index for catalysts in real applications, especially in acidic environments. The IrO_2_NR shows high stability towards the OER (Fig. 3c). The C-IrO_2_ and C-Ir/C lose their activity after 50000 s at 10 mA cm^–2^, while the activity loss of the IrO_2_NR is negligible, which is in accordance with the performance of the IrO_2_NS. The overpotential only increases by ~1.6% for the IrO_2_NR after 500000 s in 0.5 M H_2_SO_4_. The stability of the IrO_2_NR catalyst is tested at large current densities, including 50 mA cm^–2^ and 100 mA cm^–2^, as shown in Supplementary Fig. 30a. The results indicate the long-term durability of the IrO_2_NR. A descriptor of the S-number introduced by Geiger et al.^18^ is an efficient parameter for evaluating the stability of a catalyst. The S-number is defined as the number of produced oxygen molecules (n(O_2_)) per number of dissolved iridium ions (n(Ir)) for OER catalysts. IrO_2_NRs at different current densities (10, 20, 50 and 100 mA cm^–2^) and at different applied potentials (1.40 1.45, 1.50 and 1.55 V vs. RHE) are tested for 3600 s, respectively, and the S-number is calculated as shown in Supplementary Fig. 30b and listed in Supplementary Table 6. The IrO_2_NR provides more stability than other reported iridium-based OER catalysts (Supplementary Fig. 31)^18,41,46–48^. The morphology, crystal structure and element state of the IrO_2_NR after the stability test are shown in Supplementary Fig. 32 to evaluate its stability. The layered structure of the IrO_2_NR is maintained, as confirmed by the XRD pattern (Supplementary Fig. 32a). The SEM and TEM images in Supplementary Fig. 32b, c reveal that the nanoribbon structure of the IrO_2_NR does not change. The Ir and O elements are uniformly dispersed in the IrO_2_NR (Supplementary Fig. 32d–f) with the new phase confirmed by the HRTEM image in Supplementary Fig. 32g. XPS spectra of the IrO_2_NR (Supplementary Fig. 32h, i) indicate that the Ir 4 *f* peak in the IrO_2_NR maintains the +4 oxidation state. The thickness of the IrO_2_NR remains in the range of about 6.0 to 12.0 nm, as confirmed by AFM in Supplementary Fig. 33. These data all demonstrate the high stability of the IrO_2_NR.

### Theoretical analysis of the OER on IrO_2_NRs

**Fig. 4:**
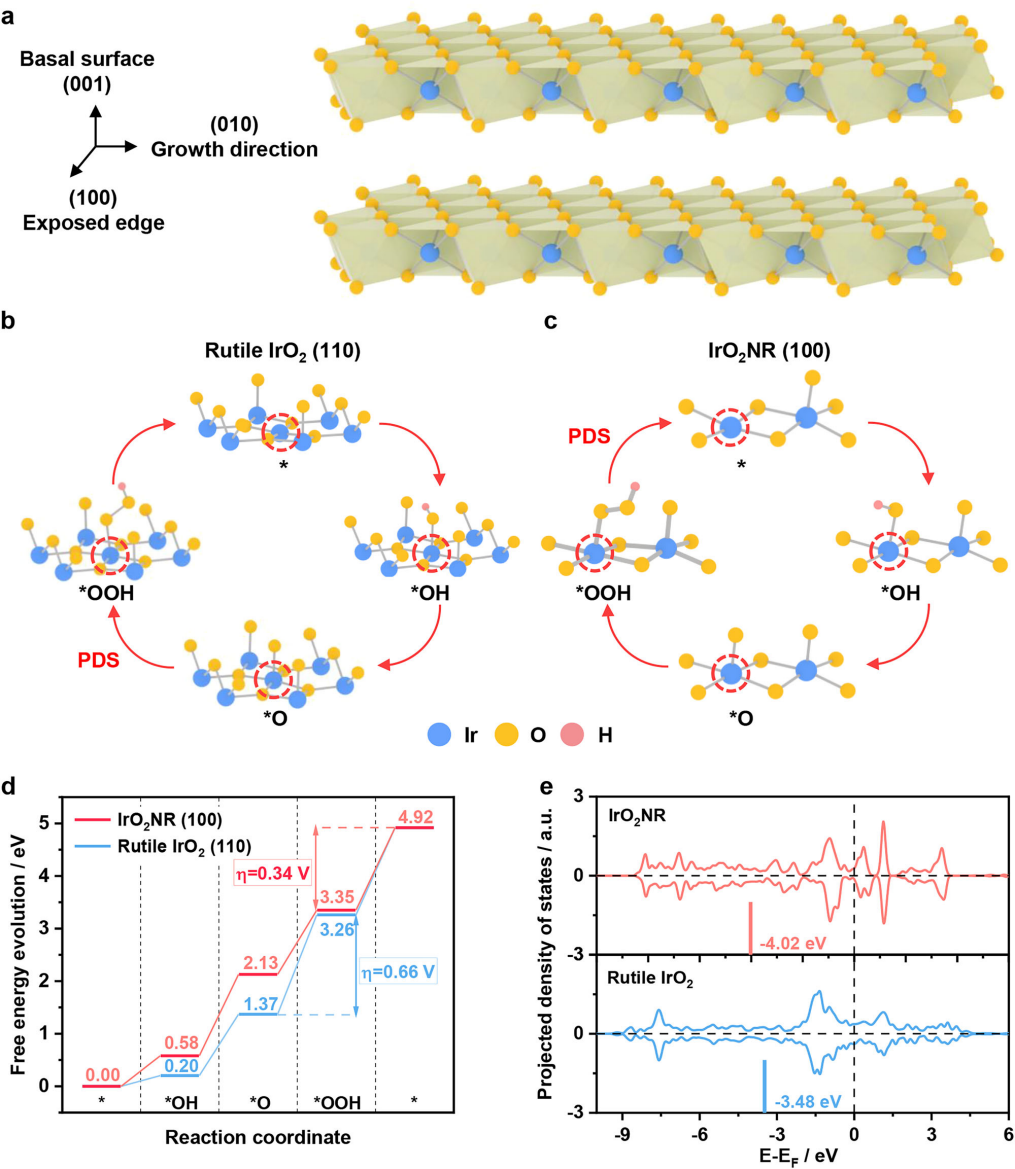
Theoretical analysis of the OER for the IrO_2_NR. **a** Crystal structure of the IrO_2_NR with an exposed edge for the (001) plane during theoretical calculation. The reaction mechanisms of the OER over the (**b**) Rutile IrO_2_ (110) and (**c**) IrO_2_NR (100) samples. **d** Corresponding 4e^-^ thermodynamic diagram of free energy evolution. **e** Comparison of the *d*-orbital distribution of the Ir atoms in the Rutile IrO_2_ and IrO_2_NR samples. Blue, yellow, and pink balls represent Ir, O and H elements, respectively.

To reveal the underlying origin of the distinguished OER performance, we conduct density functional theory (DFT) calculations to investigate the OER processes on IrO_2_NR. Here, a theoretical IrO_2_NR structure model is constructed according to the experimental results, in which the (010) direction is its growth direction. As shown in Fig. 4a, a series of theoretical calculations suggest that the (100) surface contributes to the main OER active sites of the IrO_2_NR, while that of the conventional Rutile IrO_2_ is located on the (110) surface. During the DFT calculation process, the active Ir atoms are exposed. For Rutile IrO_2_ (110), the coordinatively undersaturated Ir site is the reactive site to catalyse water into oxygen, and it does the same to the IrO_2_NR. The main difference originates from the different geometric configurations of the Ir-O octahedron. An edge-edge sharing mode of IrO_2_NR, instead of the edge-corner sharing mode in Rutile IrO_2_ (Fig. 4b, c and Supplementary Fig. 34), is helpful for weakening the adsorption of the OER intermediates^49^. In addition, a Pourbaix diagram is created to determine the surface termination of the IrO_2_NR under acidic conditions^50,51^. As shown in Supplementary Fig. 35, at *U* = 1.50 V vs. RHE, the fully oxygen-terminated IrO_2_ surface is the most stable exposed surface at pH = 0, which is consistent with the experimental conditions. As shown in Fig. 4d, the calculated potential determining step (PDS) of the Rutile IrO_2_ (110) surface occurs during the O-OH coupling process, namely *O + H_2_O → *OOH + H^+^ + e^-^, with a maximum free energy barrier of 1.89 eV, while the energy change at the PDS of O_2_ formation (*OOH → * + O_2_ + H^+^ + e^-^) for the IrO_2_NR decreases to 1.57 eV. Clearly, the obtained theoretical overpotential (η) of 0.34 V for IrO_2_NR is lower than that of for Rutile IrO_2_ (110) (0.66 V). The detailed electronic properties of the Ir atoms in the Rutile IrO_2_ and the IrO_2_NR are further analysed. As shown in Fig. 4e and Supplementary Figs. 36 and 37, the exposed Ir atoms of IrO_2_NR have lower *d* band (or higher O 2*p* band) centre than those of Rutile IrO_2_ during the OER cycles. This phenomenon contributes to the weak adsorption of the OER intermediates (Supplementary Fig. 38), self-adjusting the four-electron OER processes to a balanced free energy profile with a low overpotential. As reported, Rutile IrO_2_ tends to strongly bind O-based intermediates and results in low OER activity due to its high *d* band centre (or lower O 2*p* band)^52–54^. In our system, IrO_2_NR has a low *d* band centre, which may mainly contribute to its high OER activity.

## Discussion

Layered metal oxides belong to a unique class of functional materials and have great potential in a broad range of applications. Most recent reports focus on two-dimensional (2D) layered materials with nanosheet morphology. However, it should be noted that the nanoribbon layered materials offer a number of advantages similar to or even better than those of 2D nanosheet materials, such as a high surface area, the facilitation of electrical transport, and a natural geometry for in situ probing. Therefore, combining the advantages of the nanoribbon architectures and layered structures may have a significant impact on future applications. Through rational design and fabrication techniques, this combination may lead to an unusual phase that does not exist in bulk materials, thus generating new active sites for electrocatalytic energy conversation reactions. In this study, we obtain a metastable metal oxide with a nanoribbon morphology and discover the essential reason for its OER activity. In this case, IrO_2_NR is fabricated by a strong alkaline-assisted mechano-thermal process. The structure was determined to be monoclinic phase with a space group of C2/m (12), which evolved from monoclinic K_0.25_IrO_2_. The IrO_2_NR exhibits a superior OER activity level with an overpotential of 205 mV at 10 mA cm^–2^ in 0.5 M of H_2_SO_4_, which is 98 mV less than that of C-IrO_2_. Monoclinic IrO_2_NR shows a very high stability. The high activity level can be maintained for 500000 s during the chronopotentiometry test. In addition to the greater number of exposed active sites at the edges, the Ir atoms in monoclinic IrO_2_NR have a lower *d* band energy level than that of rutile phase IrO_2_, leading to a weaker adsorption of *O in the OER intermediates and to the self-adjusting of the four-electron OER processes to a balanced free energy profile with a low overpotential. With the rapid development of the fields of surface science, nanoscience and nanotechnology, as well as the progress of theoretical research, well-defined metal oxide ribbon nanostructures will pave the way for the design of the next-generation of solid catalysts and provide a deep understanding of structure-activity relationships.

## Methods

### Chemicals

Iridium trichloride (IrCl_3_, 99.9%) and Nafion solution (5 wt%) were obtained from Alfa Aesar Co. Potassium hydroxide (KOH, 99%) and isopropanol (99.8%) were purchased from Sinopharm Chemical Reagent Co. Commercial iridium oxide (C-IrO_2_, 99%, rutile phase) was purchased from Aladdin Chemical Reagent Co. Commercial Ir/C (C-Ir/C with Ir of 20 wt%) was purchased from Premetek Co. The reagents used were of analytical grade.

### Synthesis of layered IrO_2_NR

First, an ethanol solution of IrCl_3_ (3 mg mL^–1^, 100 mL) and KOH aqueous solution (KOH, 9 M, 300 mL) were stirred continuously at 150 °C in a Teflon mortar to form a uniform dark blue slurry. Then, the slurry was transformed into a homemade mechano-thermal corundum reactor that was fixed in a muffle furnace. The reactor was heated to different temperatures (300–700 °C) by stirring for 2 h. After cooling to room temperature, the samples were washed with double-distilled water and dried by lyophilization. When the heating temperature was 700 °C, the IrO_2_NR samples were obtained. The fabrication process is schematically shown in Supplementary Fig. 1.

### Material characterization

The morphologies of the samples were observed by scanning electron microscopy (SEM) on a Zeiss Supra 55 with an accelerating voltage of 10 kV. Transmission electron microscopy (TEM), high-resolution TEM (HRTEM), high–angle annular dark–field scanning TEM (HAADF–STEM) and elemental mapping images were further obtained by a TALOS transmission electron microscope with an accelerating voltage of 200 kV. Scanning transmission electron microscopy (STEM) images were collected on a fifth order aberration-corrected transmission electron microscope (JEOL ARM200CF) at 80 kV. X-ray diffraction patterns were collected by an X-ray powder diffractometer (XRD; Philips X’pert PRO MPD) equipped with Cu Kα radiation (*λ* = 0.15406 nm). The IrO_2_NR sample was laid on the surface of the silicon substrate and measured by powder diffractometer. The silicon substrate has no diffraction peaks. The chemical compositions of the catalysts were measured by X-ray photoelectron spectroscopy (XPS; Kratos AXIS UltraDLD) using Al Kα radiation (1486 eV). The measured binding energies were corrected based on the C 1 *s* energy at 284.6 eV. The surface topographic height of IrO_2_NR was measured via atomic force microscopy (AFM; Bruker Dimension Icon). Synchrotron X-ray absorption spectroscopy data were collected at Shanghai Synchrotron Radiation Facility (SSRF, 14 W). Ir L-edge X-ray absorption spectroscopy (XAS) was conducted in transmission mode using the Shanghai Synchrotron Radiation Facility (SSRF, 14 W), China. Pressed pellet specimens were made with mixtures of Ir-based sample powders with boron nitride. The absorption edge jump was optimized from 0–0.5. Energy calibration was conducted by using standard metal (Pt) foil. The reference spectra were used to align the sample spectra to rule out any systematic energy drifts while performing the measurements. The obtained XAS data were analysed using Athena software according to standard procedures using the Demeter program package (Version 0.9.26). The BET specific surface areas were characterized by an American Micromeritics ASAP-2020 porosimeter. The oxygen produced during the OER process was detected by a gas chromatograph (GC Agilent 7890B).

### Electrochemical measurements

All OER experiments were conducted on a CHI 760D electrochemical workstation with a standard three-electrode system. A modified glassy carbon electrode (GCE) and an SCE were chosen for the working electrode and the reference electrode, respectively. GCE has a mirror-like smooth surface with the diameter of 3 mm. A carbon rod (cylindrical carbon material with 3 mm in diameter and 5 cm in length) was selected as the counter electrode.

The catalyst solution was prepared as follows: 4 mg of the catalyst was added to the mixed solution (900 μL of isopropanol and 100 μL of 0.5 wt.% Nafion solution) and ultrasonicated to form a homogenous ink. A 4 μL dispersion was dropped on the surface of the GCE (mass loading: 169.5 mg cm_Ir_^-2^ for IrO_2_ and 45.3 mg cm_Ir_^-2^ for Ir/C) and dried naturally, which was the modified GCE as the working electrode for electrochemical testing. The linear sweep voltammetry (LSV) curves for OER tests were analysed in O_2_-saturated 0.5 M H_2_SO_4_ with 95% *i*R correction. The value of compensation resistance (*R*) was obtained by electrochemical workstation and then the *iR* correction of the LSV curves was by manual. The stability tests were without *iR* correction. LSV with a scan rate of 5 mV s^–1^ was conducted. The stability test was performed by collecting the electrocatalyst dropped on the carbon paper.

The calculation of the S-number was as follows: The IrO_2_NR catalyst ink (50 μL, 0.2 mg) was dropped on carbon paper (1 cm × 1 cm) and tested by the chronopotentiometry method at current densities of 10, 20, 50 and 100 mA cm^–2^ and applied potentials of 1.40, 1.45, 1.50 and 1.55 V vs. RHE, respectively, for 3600 s. The S-numbers were estimated from the amount of produced oxygen (n(O_2_)) by assuming 95% FE; the values were divided by the integrated amount of dissolved Ir ions (n(Ir)) under steady–state conditions. The values of n(Ir) were tested by inductively coupled plasma source mass spectrometry (ICP–MS).

The electrochemical surface areas (ECSA) of the Ir–based catalysts were determined by mercury (Hg) underpotential deposition^45^. The corresponding procedures were listed as follows: 0.8 mg catalyst was added to the mixed solution (900 μL of isopropanol and 100 μL of 0.5 wt.% Nafion solution) and ultrasonicated to form a homogenous catalyst ink. The above dispersion (4 μL) was dropped on the GCE (3 mm in diameter) and dried naturally for testing. CV curves were tested in 0.1 M HClO_4_ solution containing 1 mM mercury nitrate with a potential range from -0.3–0.7 V vs Ag/AgCl. The scan rate was 100 mV s^–1^. The corresponding calculation equation was as follows:

1 $${{{{{\rm{ECSA}}}}}}=\frac{Q}{C}=\frac{{S}_{{{{{{\rm{peak}}}}}}}}{{C}\nu }$$

where *C* is the coulombic charge of 138.6 μC cm_Ir_^–2 ^^45^. *S*_peak_ is the integral area of the adsorbed mercury in the CV curve, and *ν* is the scan rate of 100 mV s^−1^.

The turnover frequencies (TOFs) of the electrocatalysts were defined as the produced oxygen by the moles of the active sites per unit time. The TOFs were calculated as follows^55^:

2 $${{{{{{\rm{TOF}}}}}}}_{{{{{{\rm{BET}}}}}}/{{{{{\rm{ECSA}}}}}}}=\frac{(1.56\times 1{0}^{15}\,\frac{{O}_{2}/s}{{{{{{{\rm{cm}}}}}}}^{2}}{{{{{\rm{per}}}}}}\frac{{{{{{\rm{mA}}}}}}}{{{{{{{\rm{cm}}}}}}}^{2}})\times j}{({{{{{\rm{active}}}}}}\,{{{{{\rm{sites}}}}}})\times {A}_{{{{{{\rm{BET}}}}}}/{{{{{\rm{ECSA}}}}}}}}$$

The average active surface atoms per square centimetre of the IrO_2_NRs were calculated as follows:

3 $${{{{{\rm{Active}}}}}}\,{{{{{{\rm{sites}}}}}}}_{({{{{{{\rm{IrO}}}}}}}_{2}{{{{{\rm{NR}}}}}})}=\frac{1\,{{{{{\rm{atom}}}}}}}{(4.43\,\times \,3.14){{{{{\text{\AA }}}}}}^{2}}=7.18\times {10}^{14}\frac{{{{{{\rm{atoms}}}}}}}{{{{{{{\rm{cm}}}}}}}^{2}}$$

The average active surface atoms per square centimetre of C-IrO_2_ were calculated as follows:

4 $${{{{{\rm{Active}}}}}}\,{{{{{{\rm{sites}}}}}}}_{({{{{{{\rm{C}}}}}}-{{{{{\rm{IrO}}}}}}}_{2})}=\frac{2\,{{{{{\rm{atom}}}}}}}{(4.498\times 3.154){{{{{\text{\AA }}}}}}^{2}\times \surd 2}=9.97\times {10}^{14}\frac{{{{{{\rm{atoms}}}}}}}{{{{{{{\rm{cm}}}}}}}^{2}}$$

The average active surface atoms per square centimetre of C-Ir/C were calculated as follows:

5 $${{{{{\rm{Active}}}}}}\,{{{{{{\rm{sites}}}}}}}_{({{{{{\rm{C}}}}}}-{{{{{\rm{Ir}}}}}}/{{{{{\rm{C}}}}}})}	=\frac{0.5\,{{{{{\rm{atom}}}}}}}{0.5\times (\surd 2/2\times 3.839\times \surd 2/2\times 3.839){{{{{\text{\AA }}}}}}^{2}\times \,\sin 60^\circ } \\ 	=15.67\times {10}^{14}\frac{{{{{{\rm{atoms}}}}}}}{{{{{{{\rm{cm}}}}}}}^{2}}$$

The Faradaic efficiency (FE) was obtained according to the following equation:

6 $${FE}=4{nF}/{It}\times 100\%$$

where *F* is the Faraday constant (96485 C mol^–1^), *n* is the number of moles of the produced oxygen, *I* is the current (A) and *t* is the reaction time (s).

### DFT calculations

All theoretical simulations were implemented under the framework of density functional theory for the geometric and electronic analyses; their concrete realization relied on the Vienna ab-initio Simulation Package in version 5.4.1^56,57^. The description of electronic exchange–correlation energy adopted the revised Perdew–Burke–Ernzerhof function due to its more accurate treatment of the surface adsorption system^58^. Constant charge model (CCM) was used during DFT calculation in neutral cells. The electronic cut-off energy was set to 520 eV and the convergence thresholds of energy and force during geometry optimization corresponded to 10^–4^ eV and –0.03 eV Å^–1^, respectively. For IrO_2_NR, we used the Gamma-centred Monkhorst-Pack 4 × 1 × 1 k-point mesh^59^, where 4-kpoints were sampled along the growth direction. As for the Rutile IrO_2_ slab model, 4-unit-thick 2×1 (110) surface was cleaved from its bulk counterpart for calculation and a 3×4×1 k-point mesh was sampled. An implicit solvation model^60^ on top of a monolayer water covered surface was constructed (Supplementary Fig. 39) for the calculation of Pourbaix diagram. We analyse the Pourbaix diagram to determine the equilibrium surface under reaction conditions, and a four-layered (100) surface was cleaved from its bulk counterpart with full oxygen-termination for further oxygen evolution processes. The free energy evaluation of the four-electron OER was based on a computational hydrogen electrode model^61,62^, in which the free energy of the electron-proton pair was approximated to half that of hydrogen at room temperature. The OER pathway follows a widely accepted adsorbate evolution mechanism as shown in Eqs. (7)-(10).

7 $${{{{{{\rm{H}}}}}}}_{2}{{{{{\rm{O}}}}}}+\ast \to {{{{{\rm{OH}}}}}}\ast \,+\,({{{{{{\rm{H}}}}}}}^{+}+{{{{{{\rm{e}}}}}}}^{-})$$

8 $${{{{{\rm{OH}}}}}}\ast \to {{{{{\rm{O}}}}}}\ast \,+\,({{{{{{\rm{H}}}}}}}^{+}+{{{{{{\rm{e}}}}}}}^{-})$$

9 $${{{{{{\rm{H}}}}}}}_{2}{{{{{\rm{O}}}}}}+{{{{{\rm{O}}}}}}\ast \to {{{{{\rm{OOH}}}}}}\ast \,+\,({{{{{{\rm{H}}}}}}}^{+}+{{{{{{\rm{e}}}}}}}^{-})$$

10 $${{{{{\rm{OOH}}}}}}\ast \to {{{{{{\rm{O}}}}}}}_{2}+\ast \,+\,({{{{{{\rm{H}}}}}}}^{+}+{{{{{{\rm{e}}}}}}}^{-})$$

The change of the Gibbs free energy (ΔG) for each intermediate is defined by ΔG = ΔE + ΔE_ZPE_ - TΔS, where ΔE_ZPE_ is the difference in zero-point energy between the adsorbed and the gas phase, and T is the temperature of 300 K and ΔS is the entropy change. The theoretical overpotential was determined by $\eta={{\max }}\left\{{G}_{i}\right\}-1.23$, where ${G}_{i}$ represents the free energy change of every OER step. The simulated coordinates for IrO_2_NR and Rutile IrO_2_ were supplied in Supplementary Notes 1 and 2 and the code input file was in Supplementary Note 3. The zero-point vibrational energies (ZPVEs) and entropic contributions were given in Supplementary Table 7.

## Supplementary information

Supplementary Information

Peer Review File

## Data Availability

The data generated in this study are provided in the Supplementary Information/Source Data file. Source data are provided with this paper.

## Source data

Source Data
