# Supplementary material for: Iridium oxide nanoribbons with metastable monoclinic phase for highly efficient electrocatalytic oxygen evolution
Source: Nat Commun. 2023 Mar 4;14:1248. doi: 10.1038/s41467-023-36833-1 (PMC9985653; doi:10.1038/s41467-023-36833-1)
Supplement: Supplementary file 1 — Supplementary Information [file 41467_2023_36833_MOESM1_ESM.pdf]

Supplementary Figures

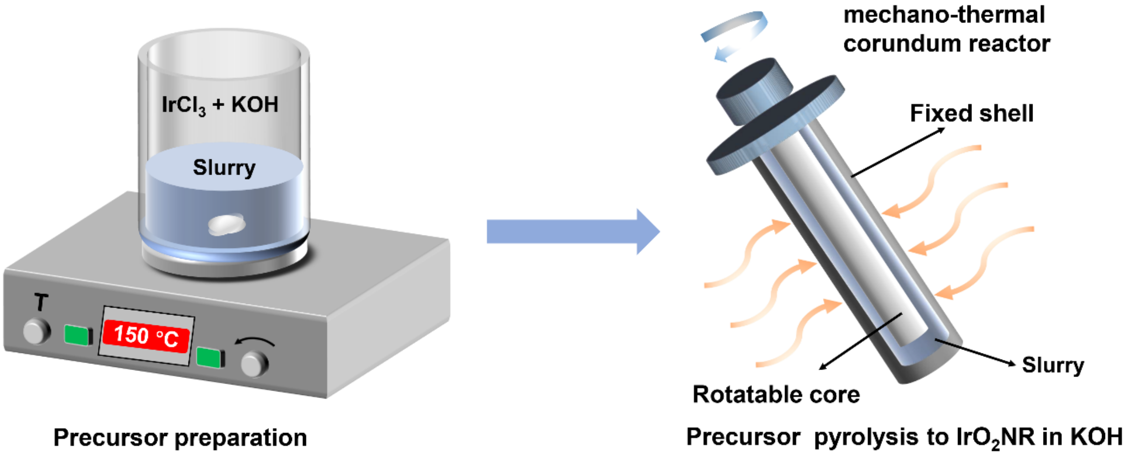

Supplementary Figure 1. The schematic molten-alkali mechanochemical reaction for synthesizing IrO_2NR. The slurry of $IrCl_3$ and KOH aqueous solution was transformed into a homemade mechano-thermal corundum reactor that was fixed in a muffle furnace. The reactor was heated to different temperatures to obtain samples.

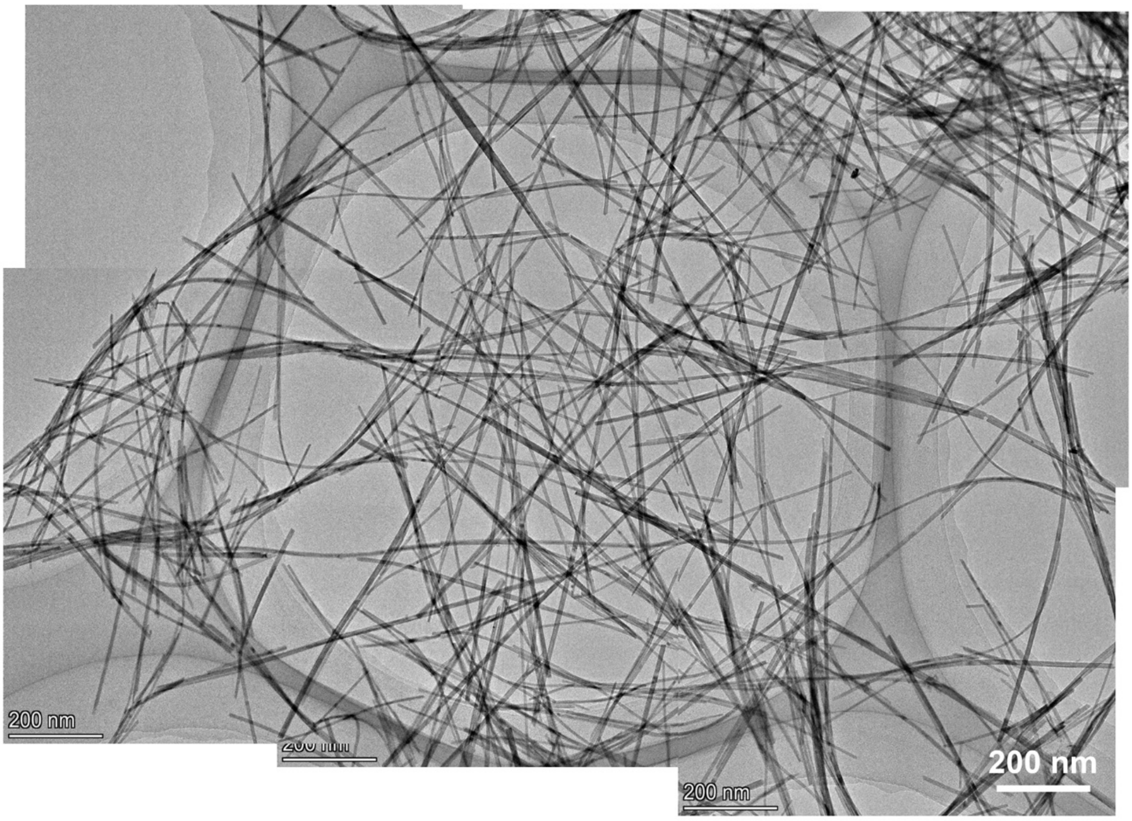

Supplementary Figure 2. TEM image of IrO_2NR. TEM images show nanoribbon morphology at a high magnification.

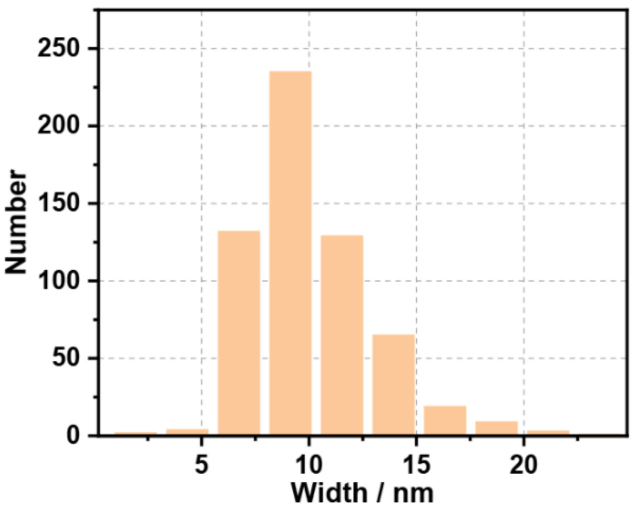

Supplementary Figure 3. Statistics histogram showing the width of IrO_2NR. The width values are obtained by 500 nanoribbons.

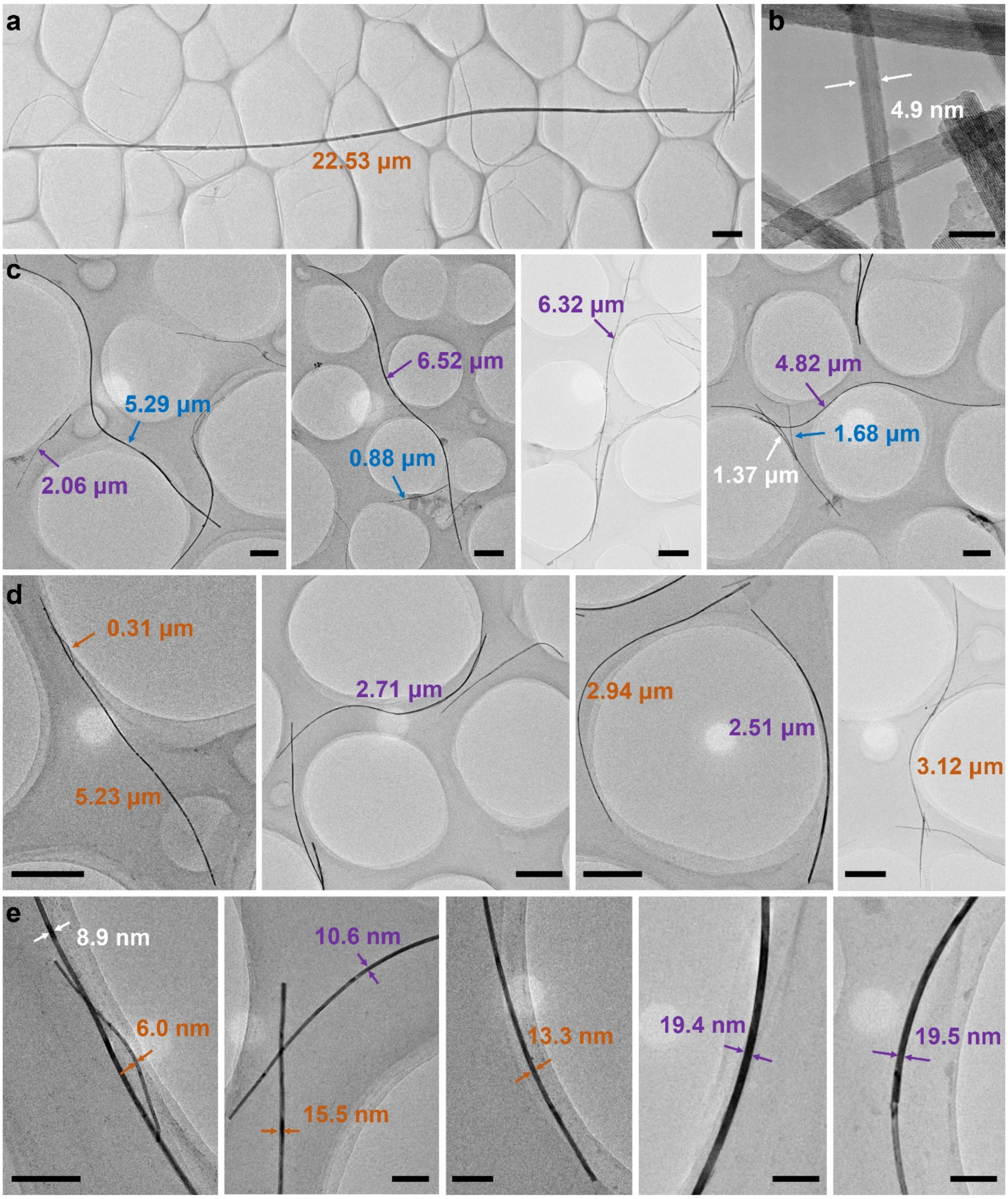

Supplementary Figure 4. Gallery of IrO_2NR. (**a**) Typical image of a long IrO_2NR. Figure bar, 1 μm; insert bar, 100 nm. (**b**) Typical image of a thin IrO_2NR. Figure bar, 10 nm. (**c**) and (**d**) IrO_2NR of different lengths. Figure bar, 500 nm. (**e**) Micrographs of IrO_2NR with different widths. Figure bar, 100 nm.

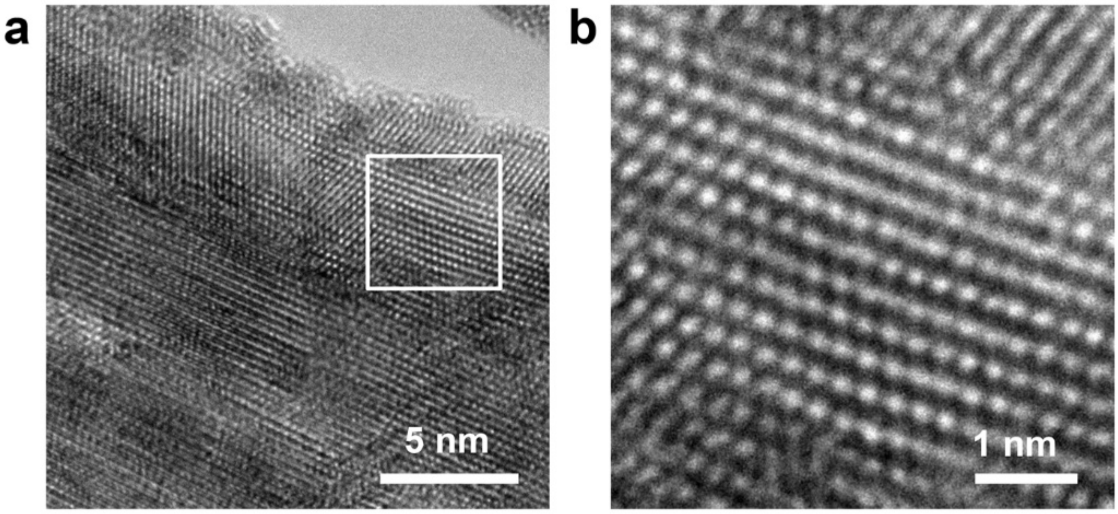

Supplementary Figure 5. Different magnification HETEM images. (**a**) Low magnification and (**b**) high magnification HRTEM images of IrO_2NR showing the lattice tension/compression.

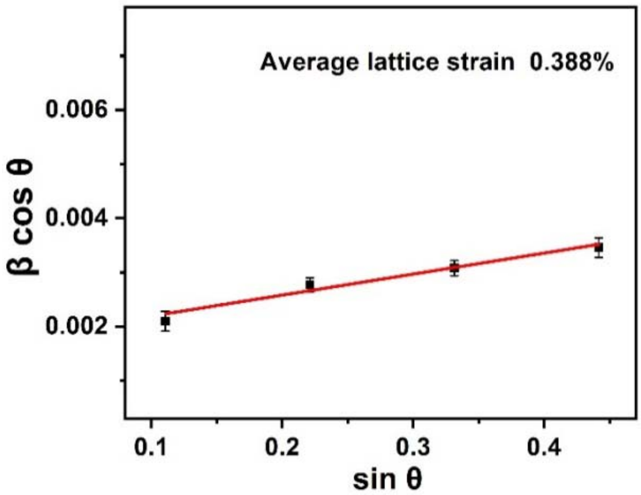

Supplementary Figure 6. Plot for Williamson-Hall analysis to calculate the lattice strain of IrO_2NR. According to the Williamson-Hall equation: $\beta \times \cos\theta = K\lambda / D + 4\varepsilon \times \sin\theta$, where β is the full width at half-maximum of the peak, θ is the Bragg angle, K is the shape factor, D is the crystallite size, and λ is the wavelength of X-ray, the lattice strain of IrO_2NR is calculated to be 0.388%. Error bars represent standard deviation, n = 3 independent replicates.

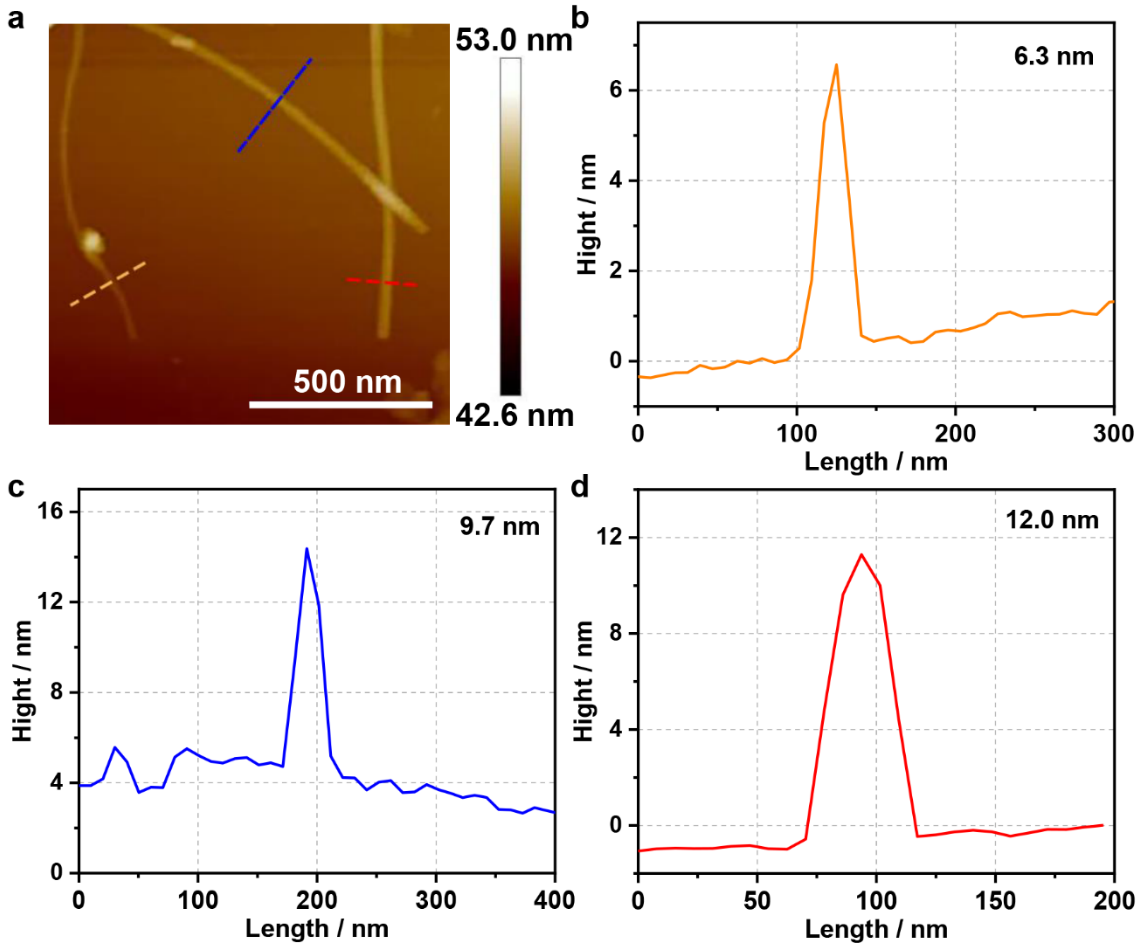

Supplementary Figure 7. Thickness of IrO_2NR. (**a**) AFM image and (**b-d**) the corresponding height profiles showing the thickness of the IrO_2NR.

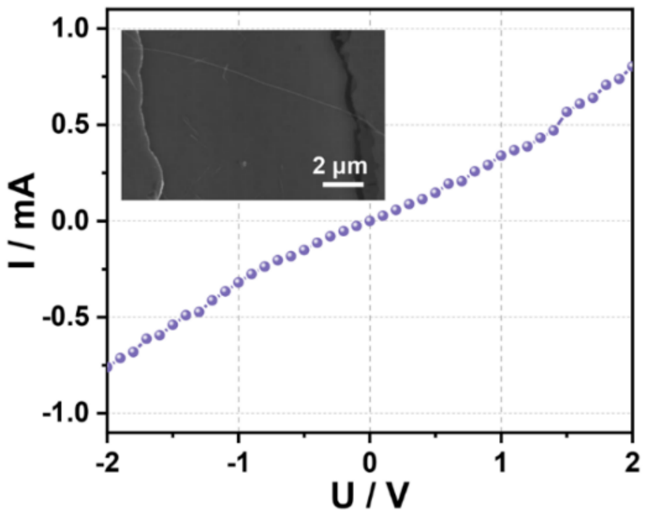

Supplementary Figure 8. I – V curve. A representative bottom-contact device was fabricated from a single IrO_2NR on an ITO substrate. Insert is the optical photograph of the device.

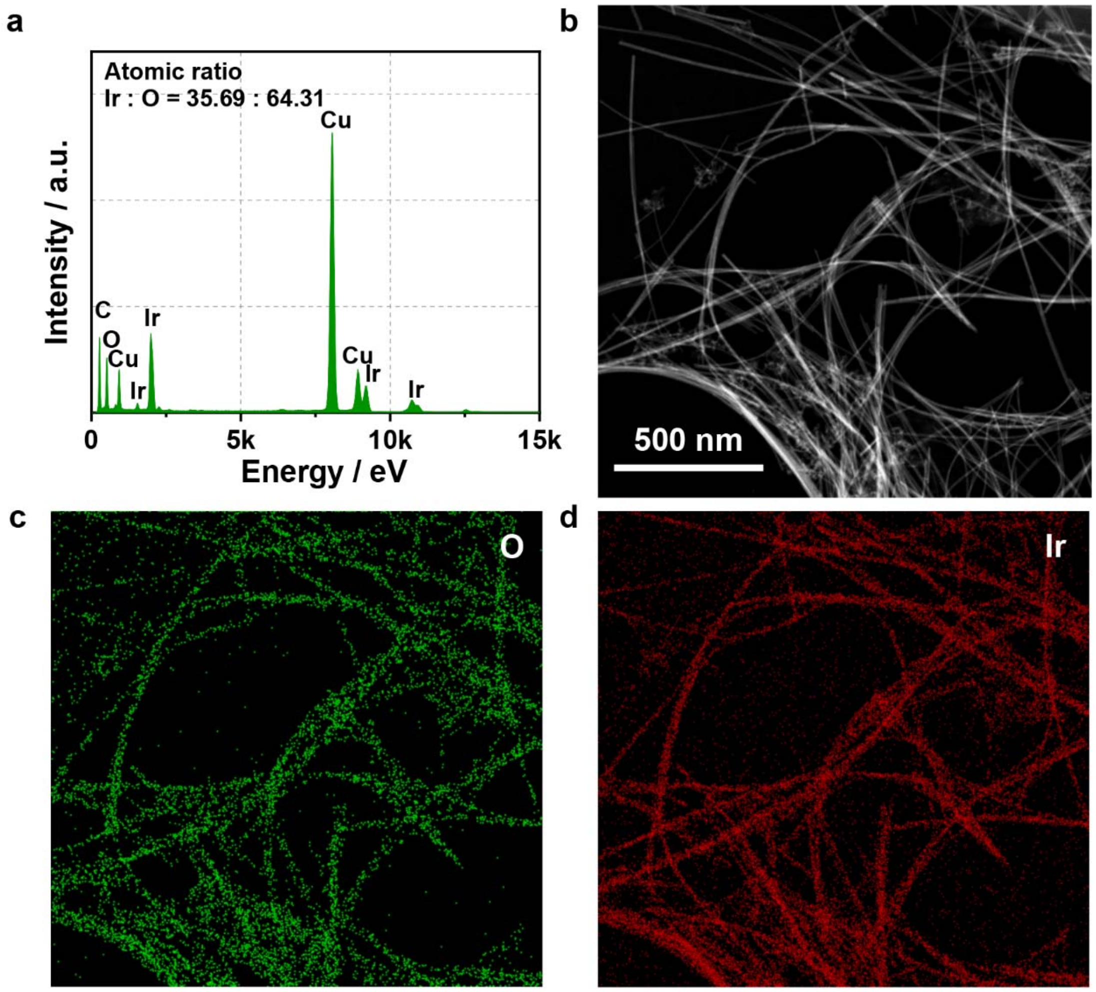

Supplementary Figure 9. Chemical composition analysis of IrO_2NR. (**a**) TEM-EDS spectrum of IrO_2NR holding on a Cu grid. (**b**) HAADF-STEM image and corresponding elemental EDS mapping image showing the distributions of (**c**) O and (**d**) Ir elements.

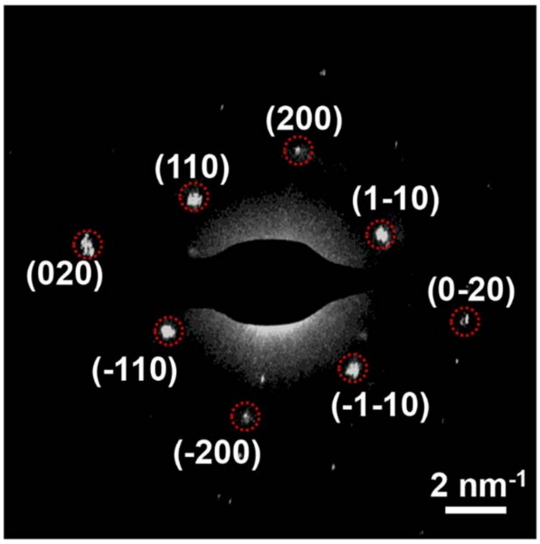

Supplementary Figure 10. SAED pattern. SAED pattern of IrO_2NR from the [001] direction.

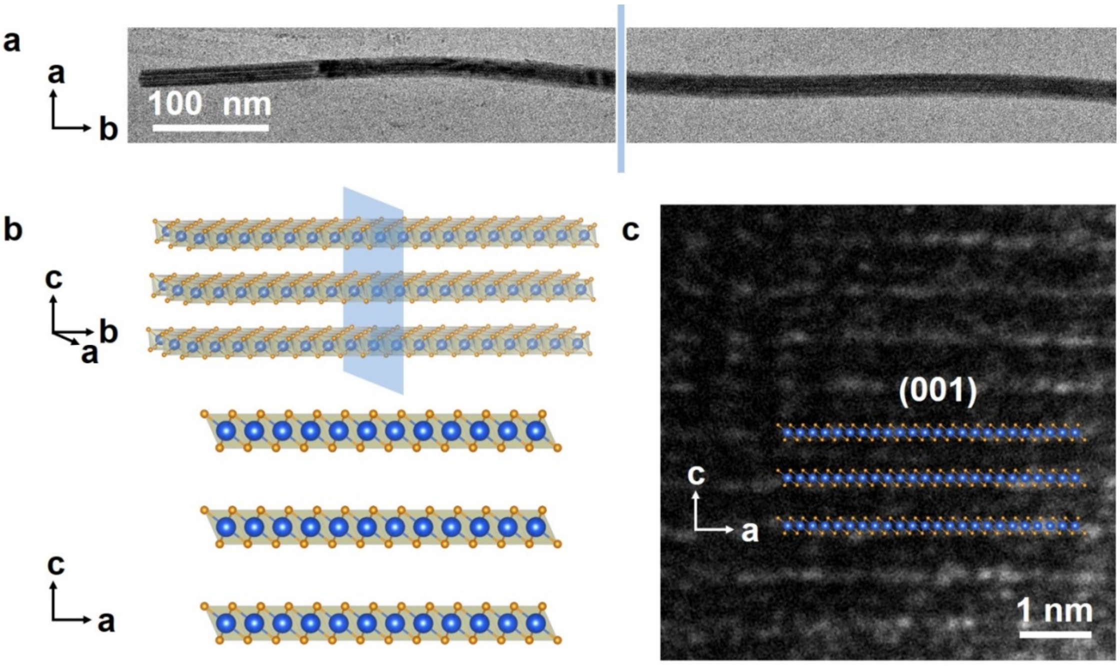

Supplementary Figure 11. Cross section of IrO_2NR. (**a**) and (**b**) Schematic showing that the IrO_2NR was embedded in epoxy resin and sliced into pieces to observe the cross section. (**c**) HRTEM image of the cross section for IrO_2NR showing the (001) plane with distance of 0.69 nm.

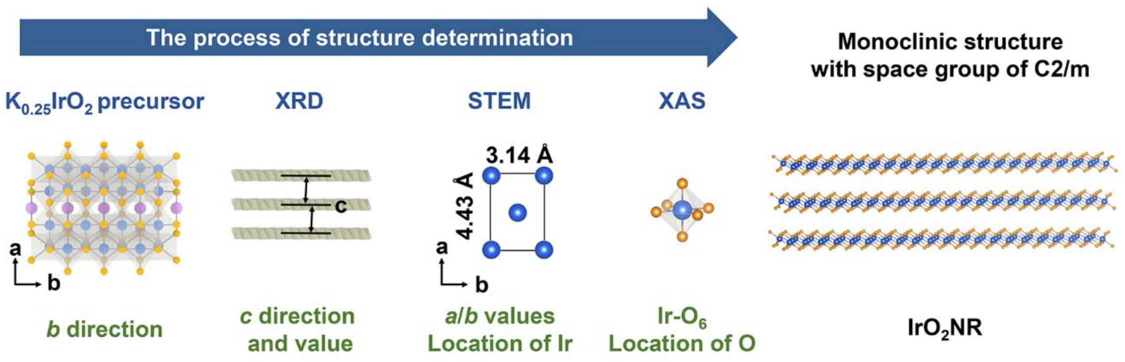

Supplementary Figure 12. Structure determination process. The schematic diagram showing the process of the structure determination of IrO_2NR.

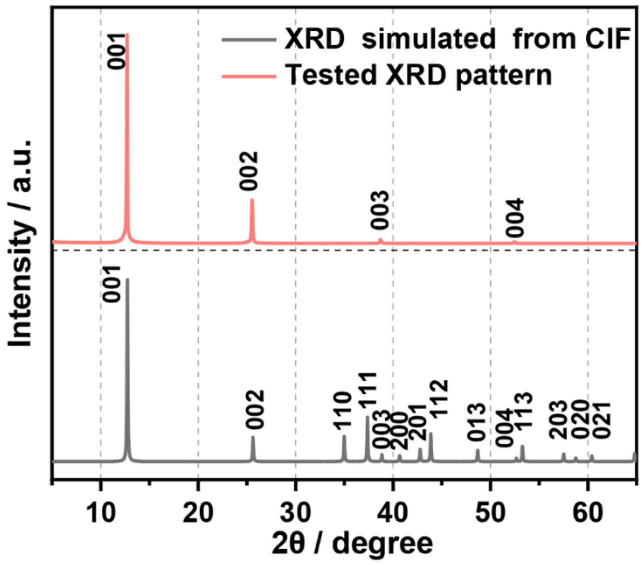

Supplementary Figure 13. XRD patterns. The simulated and tested XRD patterns of IrO_2NR.

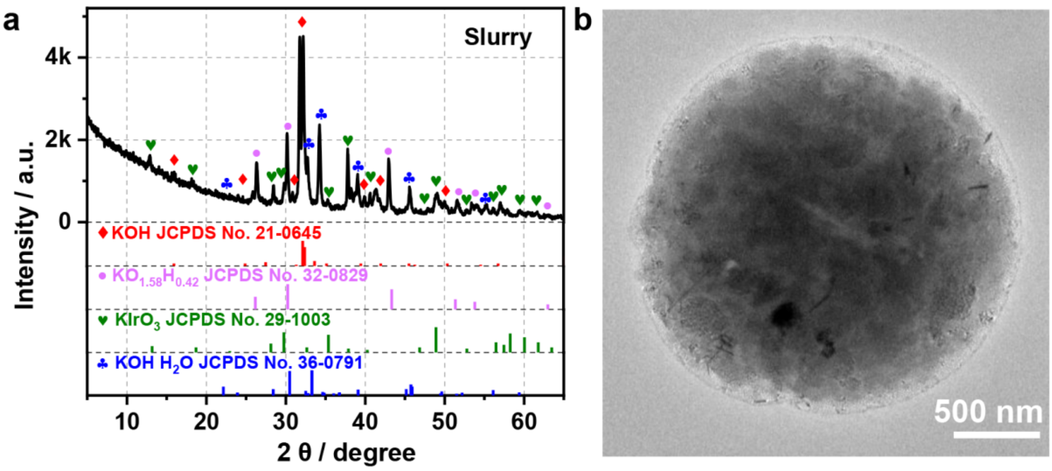

Supplementary Figure 14. Characterization of the initial slurry before washing by water. (**a**) XRD pattern and (**b**) TEM image.

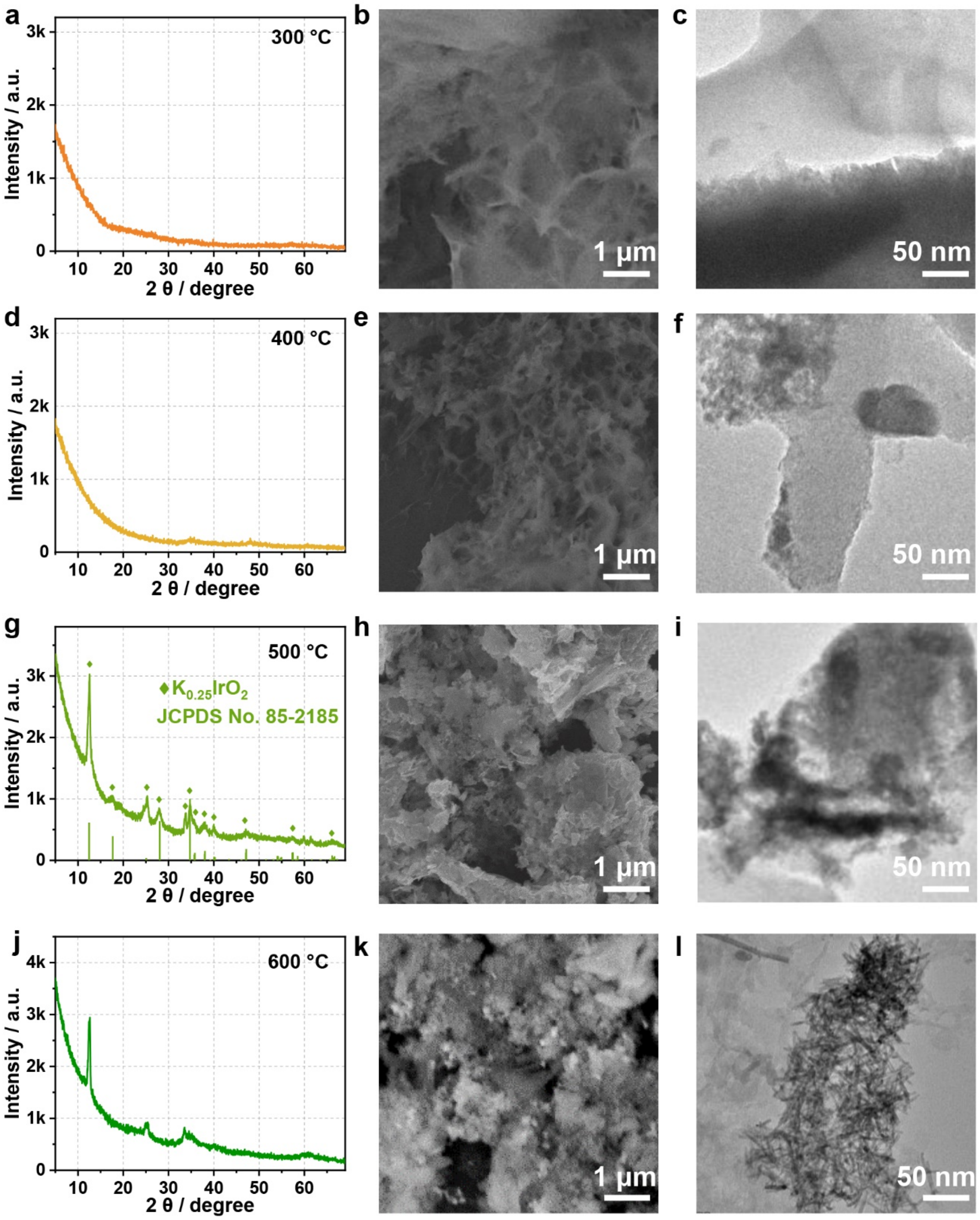

Supplementary Figure 15. The phase and morphology of samples at different reaction temperatures. (**a**) XRD pattern, (**b**) SEM image and (**c**) TEM image of sample annealed at 300 °C. (**d**) XRD pattern, (**e**) SEM image and (**f**) TEM image of sample annealed at 400 °C. (**g**) XRD pattern, indexed as $K_{0.25}IrO_2$; (**h**) SEM image and (**i**) TEM image of sample annealed at 500 °C. (**j**) XRD pattern, (**k**) SEM image and (**l**) TEM image of sample annealed at 600 °C.

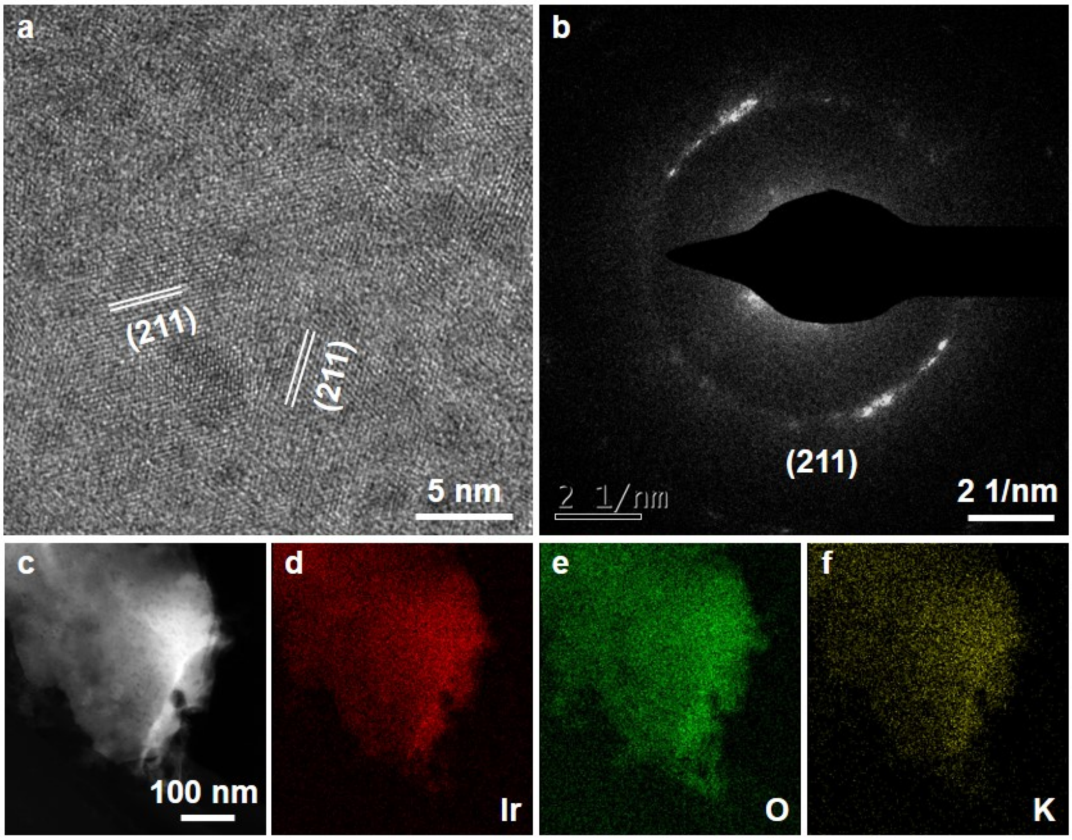

Supplementary Figure 16. Chemical composition analysis of $K_{0.25}IrO_2$ obtained at reaction temperature of 500 °C. (**a**) HRTEM image, (**b**) SAED, (**c**) HAADF-STEM image and corresponding elemental EDS mapping image showing the distributions of (**d**) Ir, (**e**) O, and (**f**) K elements.

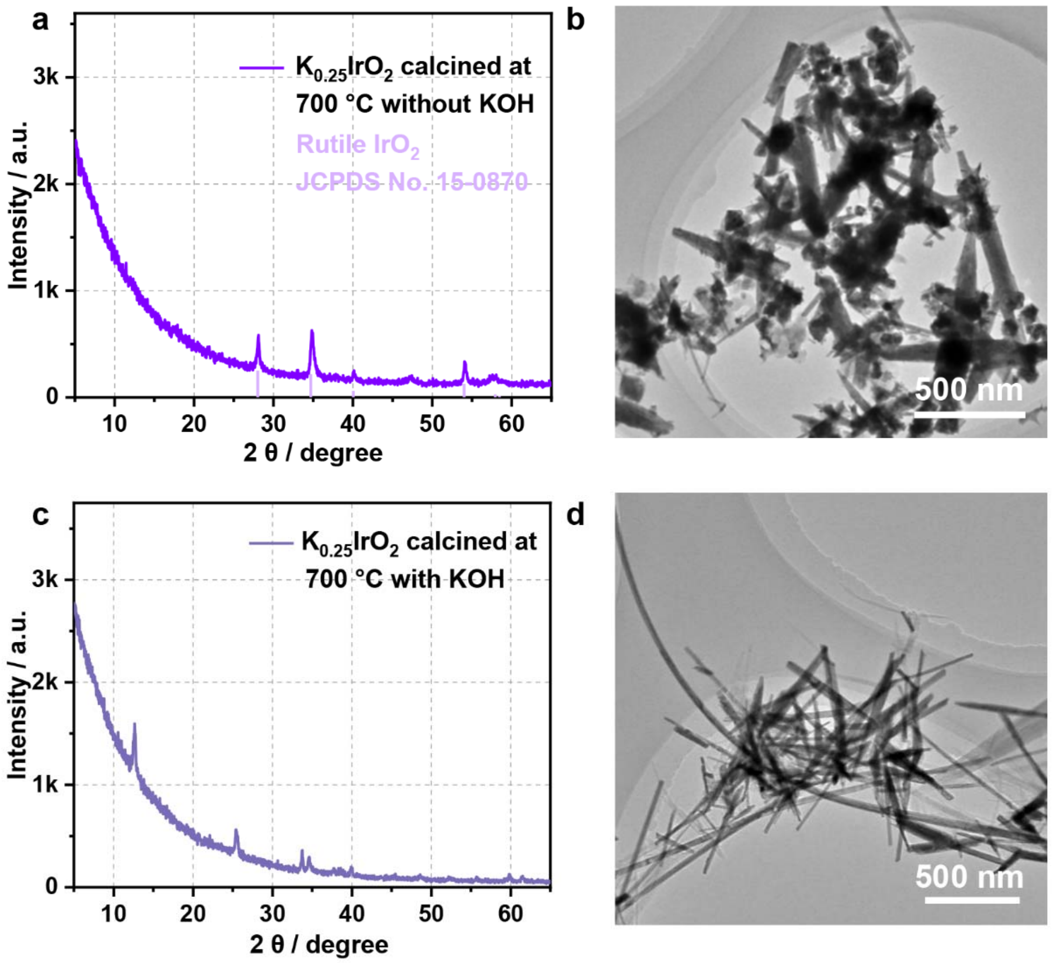

Supplementary Figure 17. The comparison experiments showing the important role of the alkaline condition. (**a**) XRD pattern of sample by calcining $K_{0.25}IrO_2$ at 700 °C for 2 h without KOH, which is indexed as Rutile IrO_2. (**b**) TEM image of the above mentioned Rutile IrO_2. (**c**) XRD pattern of the sample that calcined the mixture of $K_{0.25}IrO_2$ and KOH at 700 °C for 2 h, where the layered structure appears. (**d**) TEM image of the above mentioned sample.

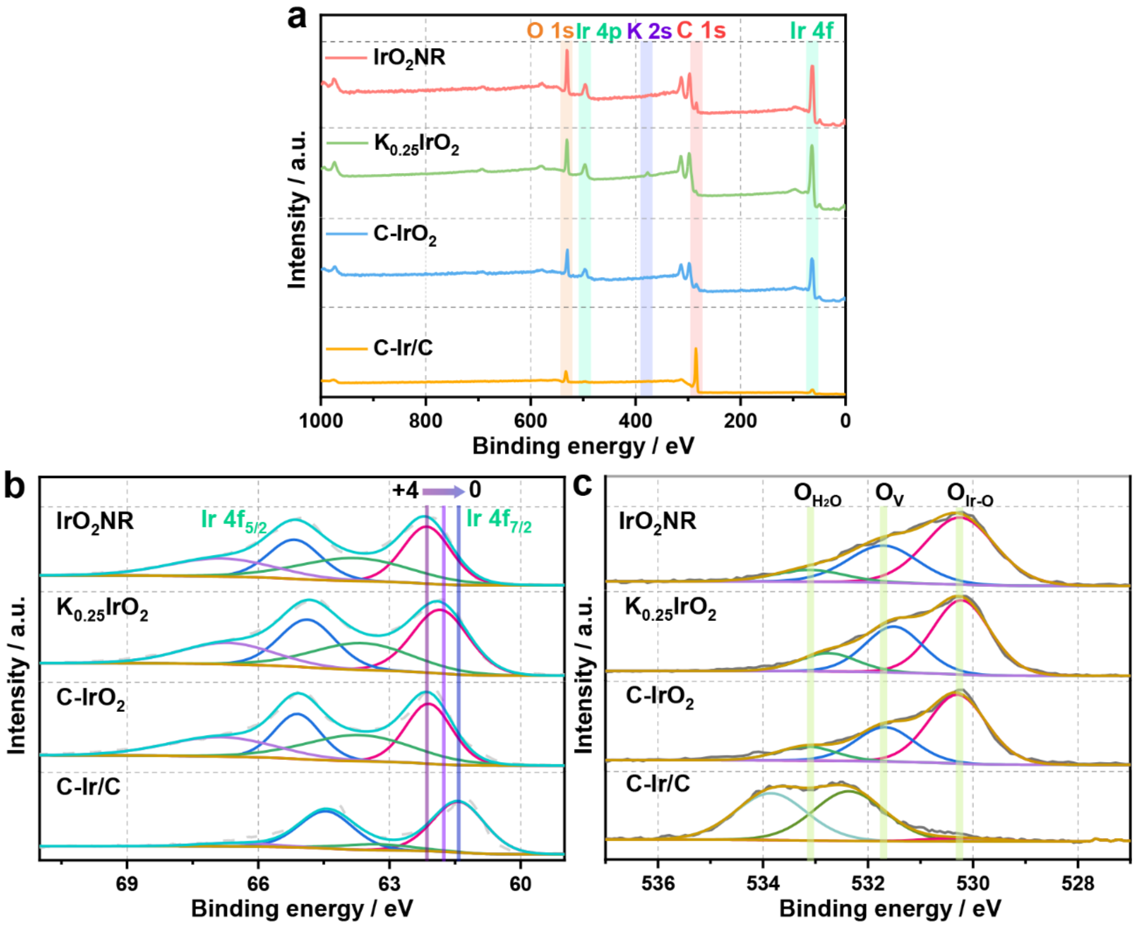

Supplementary Figure 18. XPS spectra. (**a**) Full spectra; (**b**) Ir 4*f* peaks and (**c**) O 1*s* peaks for IrO_2NR, $K_{0.25}IrO_2$, C-IrO_2 and C-Ir/C. The O 1*s* for C-Ir/C may come from the adsorbed oxygen species with carbon materials.

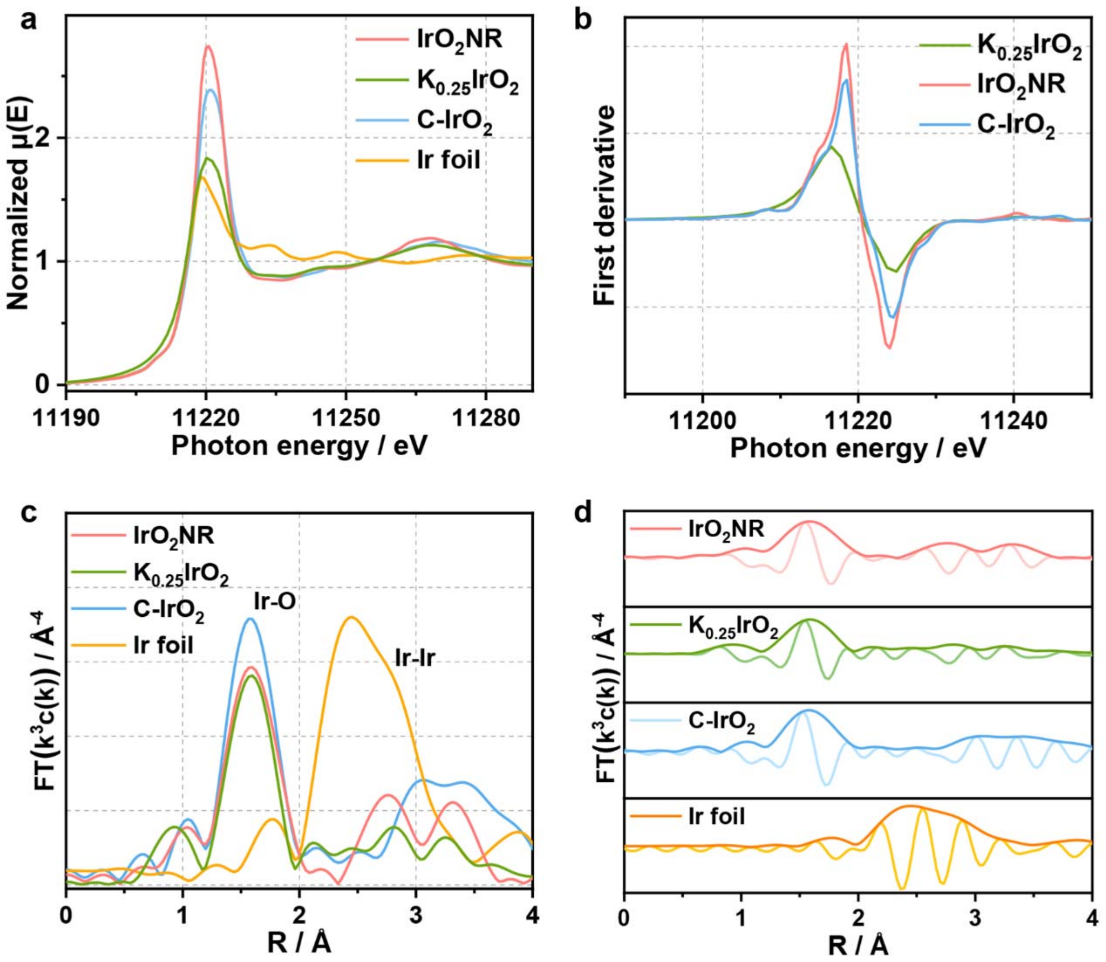

Supplementary Figure 19. XAS analysis of IrO_2NR, $K_{0.25}IrO_2$, C-IrO_2, and Ir foil. (**a**) XANES spectra for IrO_2NR, $K_{0.25}IrO_2$, C-IrO_2 and Ir foil. (**b**) The first derivative of normalized intensity for IrO_2NR, $K_{0.25}IrO_2$ and C-IrO_2 in (**a**). (**c**) FT-EXAFS spectra for IrO_2NR, $K_{0.25}IrO_2$, C-IrO_2 and Ir foil. (**d**) The stacked magnitude and imaginary parts of IrO_2NR, $K_{0.25}IrO_2$, C-IrO_2, and Ir foil.

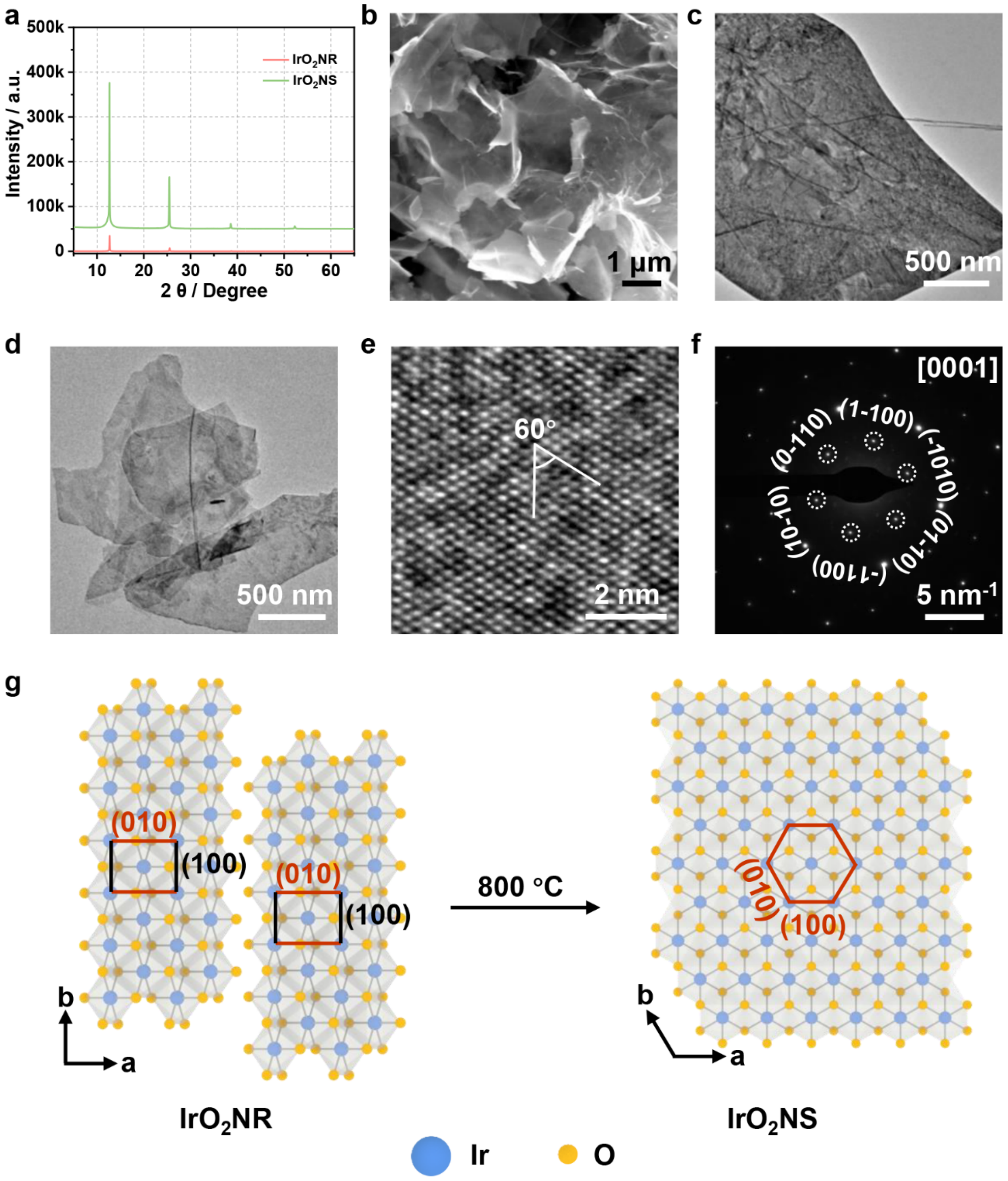

Supplementary Figure 20. The phase, morphology and formation mechanism of samples calcined at temperature of 800 °C. (**a**) XRD pattern of IrO_2NS and IrO_2NR; (**b**) SEM image of IrO_2NS; (**c**) and (**d**) TEM image of IrO_2NS, most of the sample showing nanosheet structure with several nanoribbons in it. (**e**) HRTEM image, and (**f**) SAED pattern of IrO_2NS; (**g**) Schematic diagram illustrating the structure evolution of IrO_2NR to IrO_2NS.

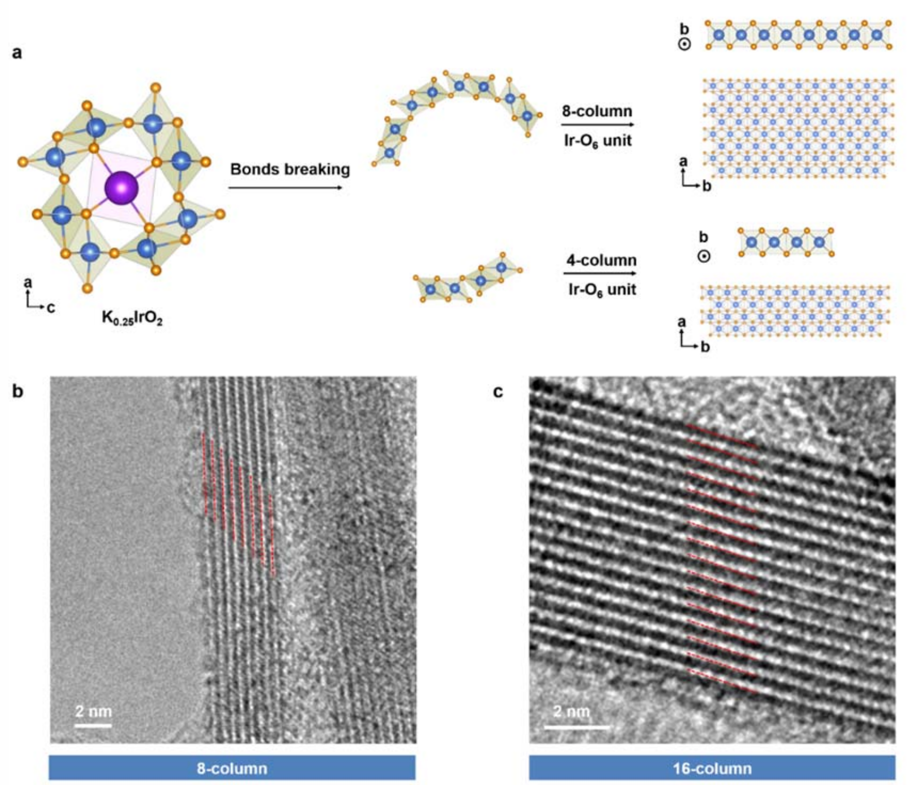

Supplementary Figure 21. The conversion of $K_{0.25}IrO_2$ to IrO_2NR. (**a**) Schematic showing that the columns of [IrO_6] subunits that perpendicular to the ***b*** direction are even numbers, taking 4 and 8 columns as examples. (b) and (c) Corresponding HRTEM images showing the even number layers of [IrO_6] subunits.

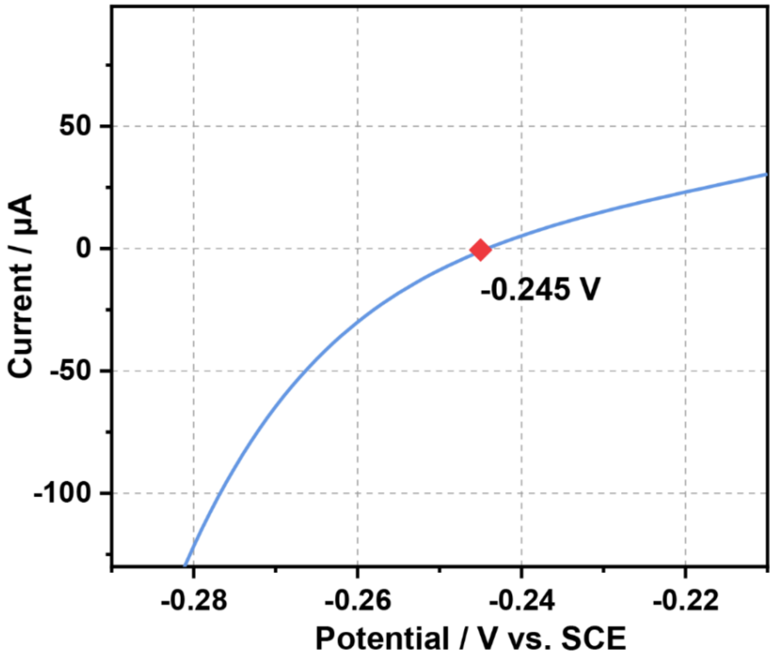

Supplementary Figure 22. Calibration of the saturated calomel electrode (SCE). Calibration of the SCE electrode with respect to reversible hydrogen electrode (RHE) in 0.5 M H_2SO_4 electrolytes bubbled with pure hydrogen gas at room temperature. Scan rate: 1 mV s^{-1}.

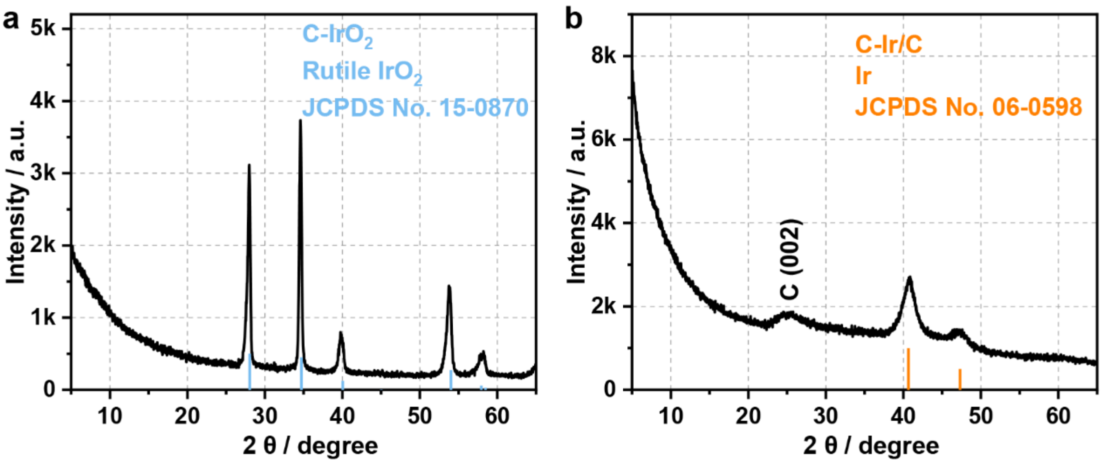

Supplementary Figure 23. XRD patterns of commercial samples. (**a**) C-IrO_2 and (**b**) C-Ir/C.

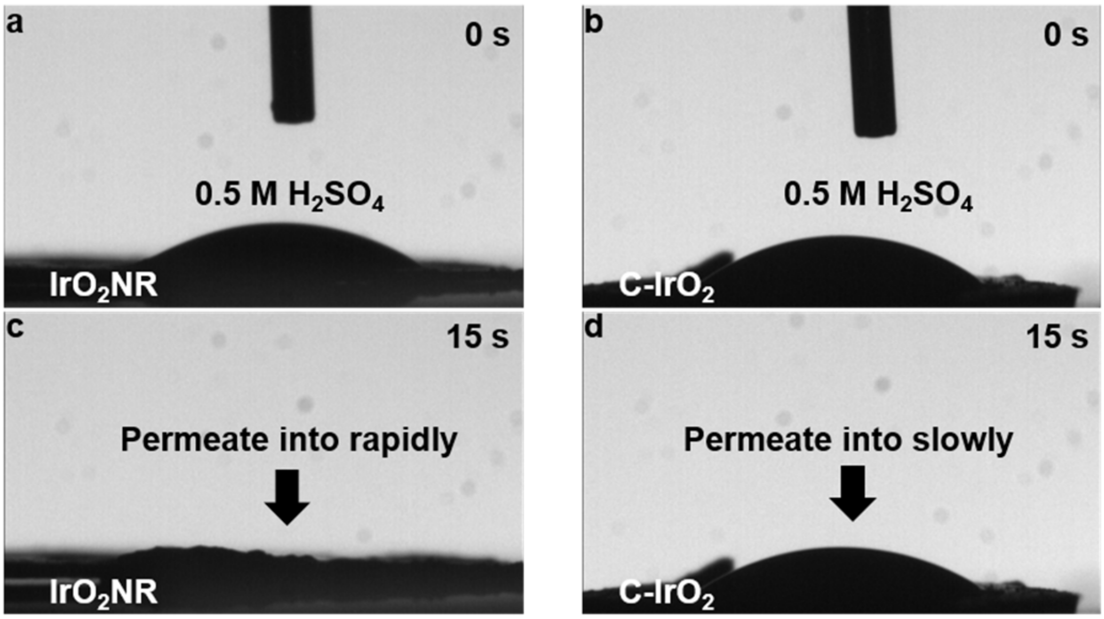

Supplementary Figure 24. The contact angles at different time. H_2SO_4 (0.5 M) droplet on IrO_2NR tablet at (**a**) 0 s and (**c**) 15 s. H_2SO_4 (0.5 M) droplet on C-IrO_2 tablet at (**b**) 0 s and (**d**) 15 s. The H_2SO_4 permeate into the IrO_2NR more rapidly than that into C-IrO_2.

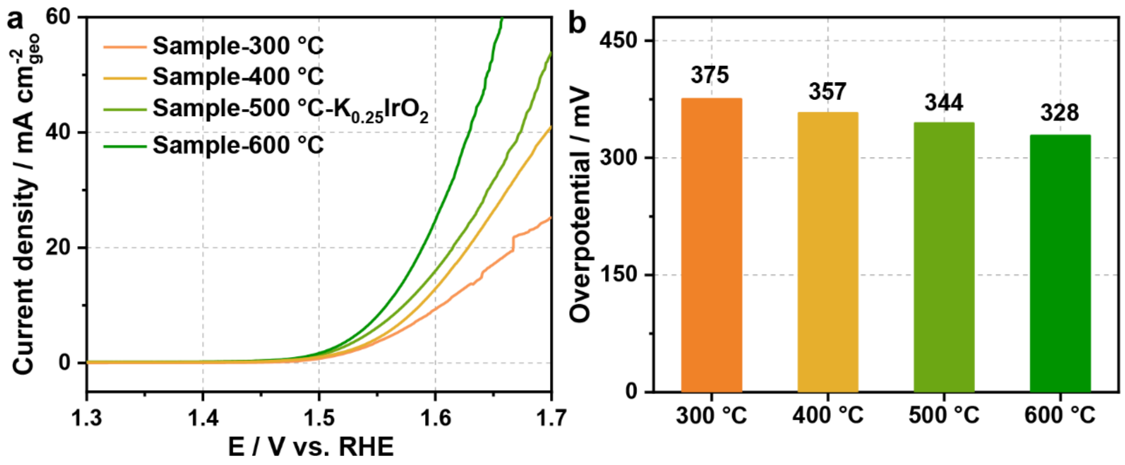

Supplementary Figure 25. OER performance of samples obtained at different reaction steps. (**a**) The LSV curves, and (**b**) histograms showing the overpotentials of corresponding samples in 0.5 M H_2SO_4.

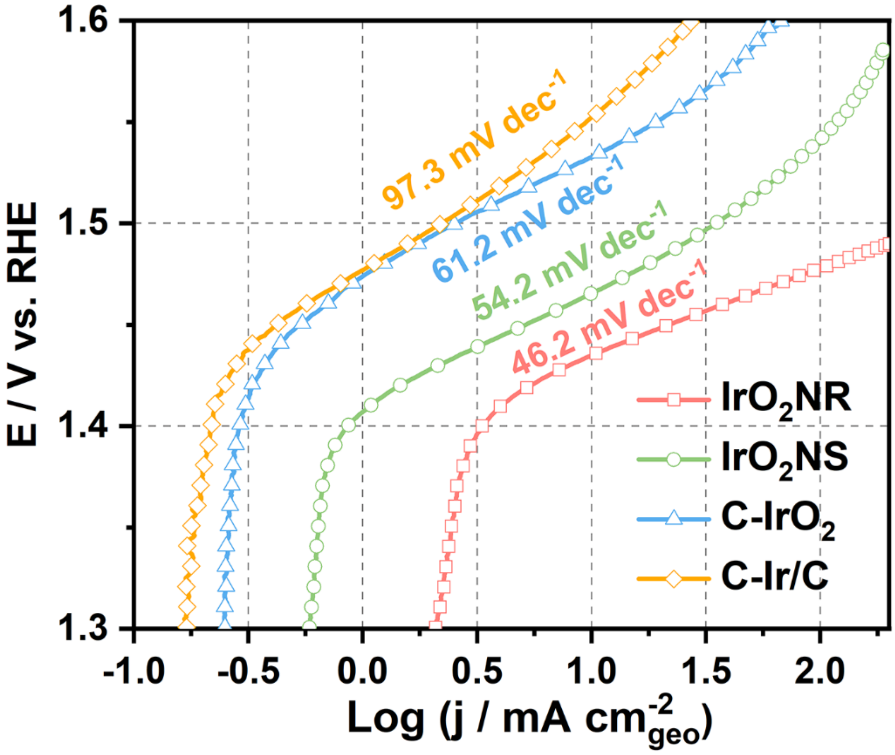

Supplementary Figure 26. Tafel plots. Tafel plots and corresponding Tafel slopes of IrO_2NR, IrO_2NS, C-IrO_2, and C-Ir/C.

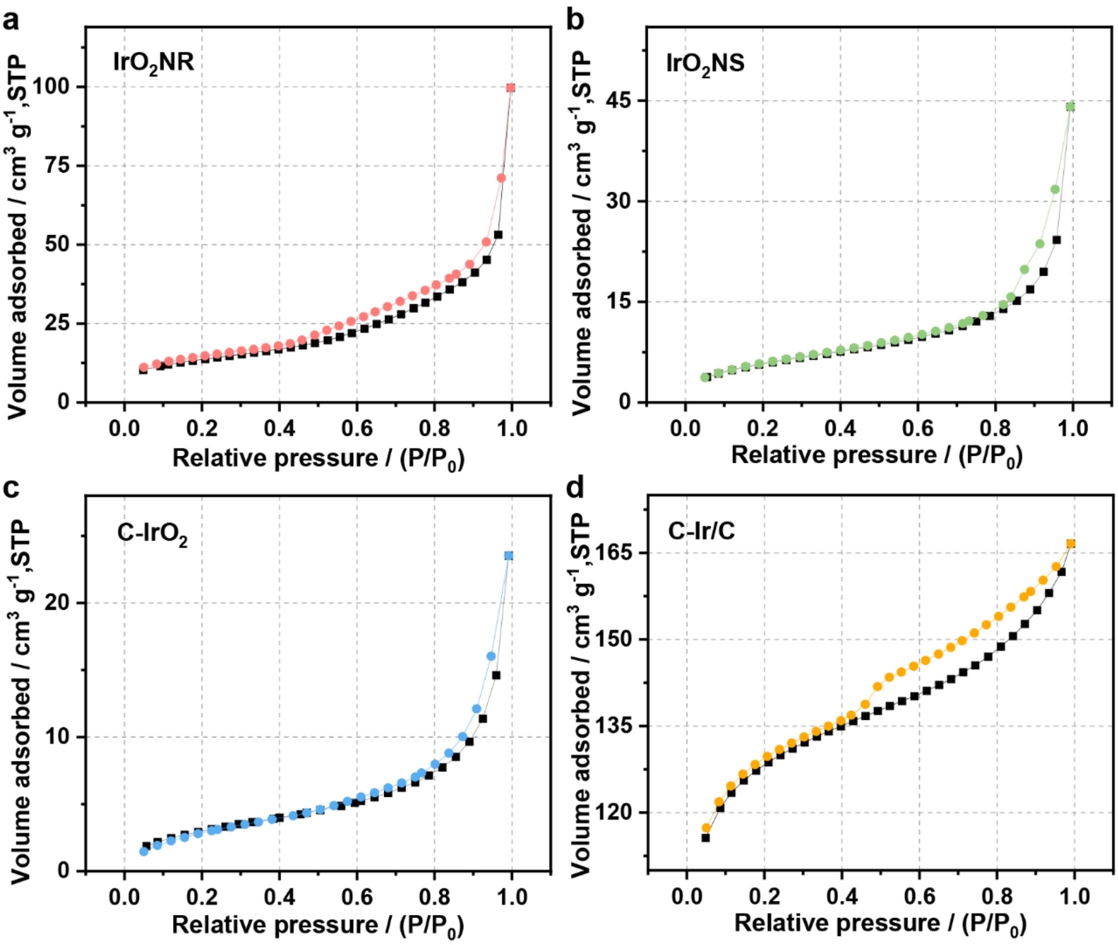

Supplementary Figure 27. BET surface area measurements. N_2 adsorption-desorption isotherms obtained for (**a**) IrO_2NR; (**b**) IrO_2NS; (**c**) C-IrO_2; and (**d**) C-Ir/C.

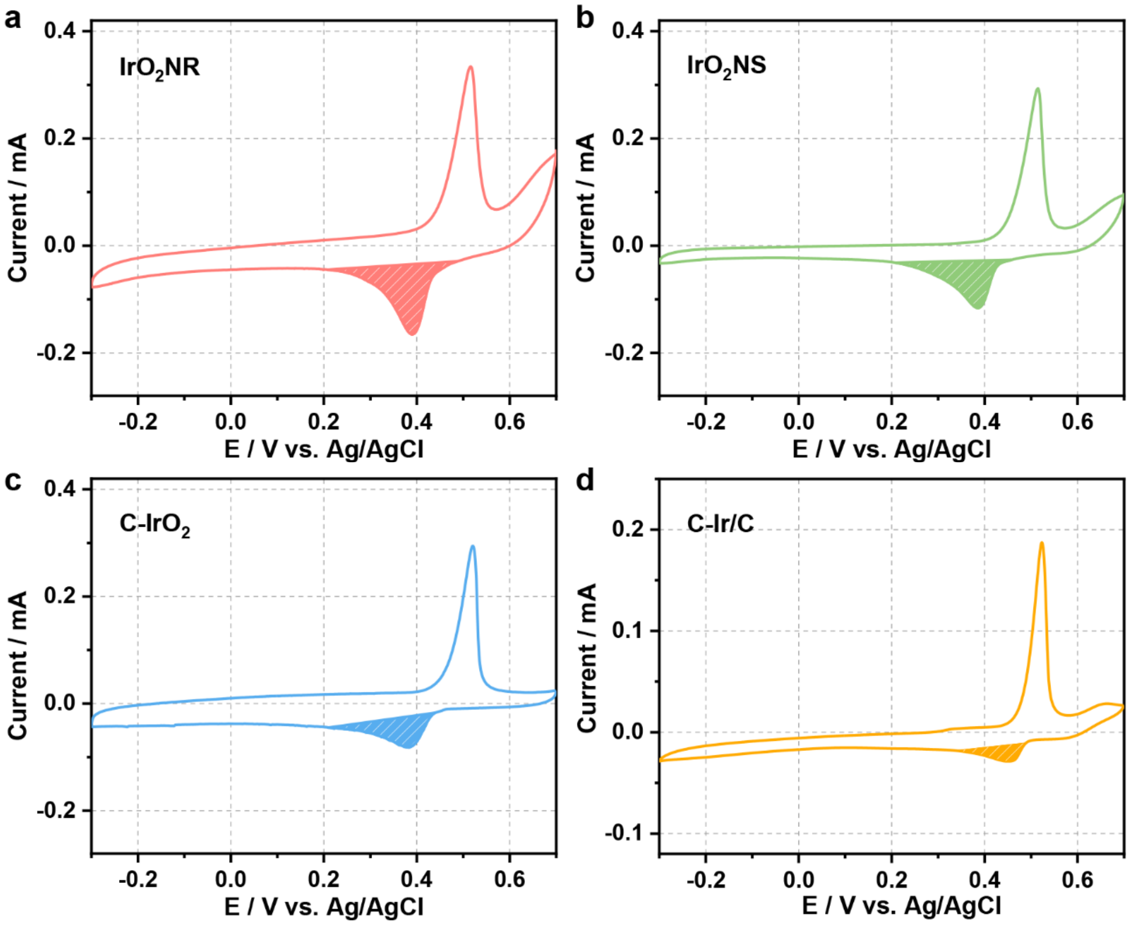

Supplementary Figure 28. ECSA measurements. The CV curves of (**a**) IrO_2NR; (**b**) IrO_2NS; (**c**) C-IrO_2; and (**d**) C-Ir/C in 0.1 M $HClO_4$ electrolyte containing 1 mM mercury nitrate at the scan rate of 100 mV s^{-1}.

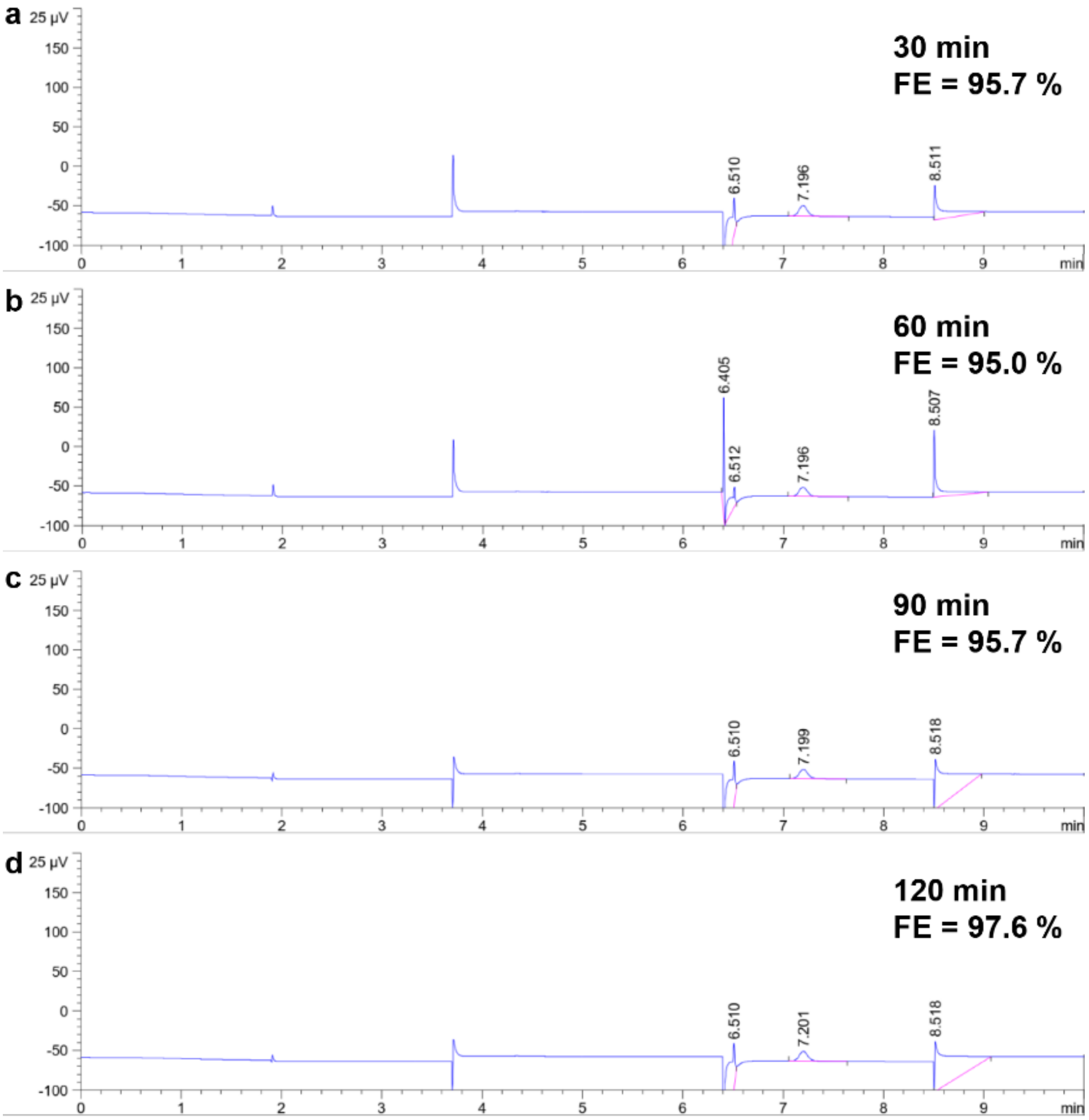

Supplementary Figure 29. Gas chromatograph data of the produced oxygen by IrO_2NR at different reaction time at a current density of 20 mA cm_{geo}^{-2}. (**a**) 30 min; (**b**) 60 min; (**c**) 90 min; and (**d**) 120 min.

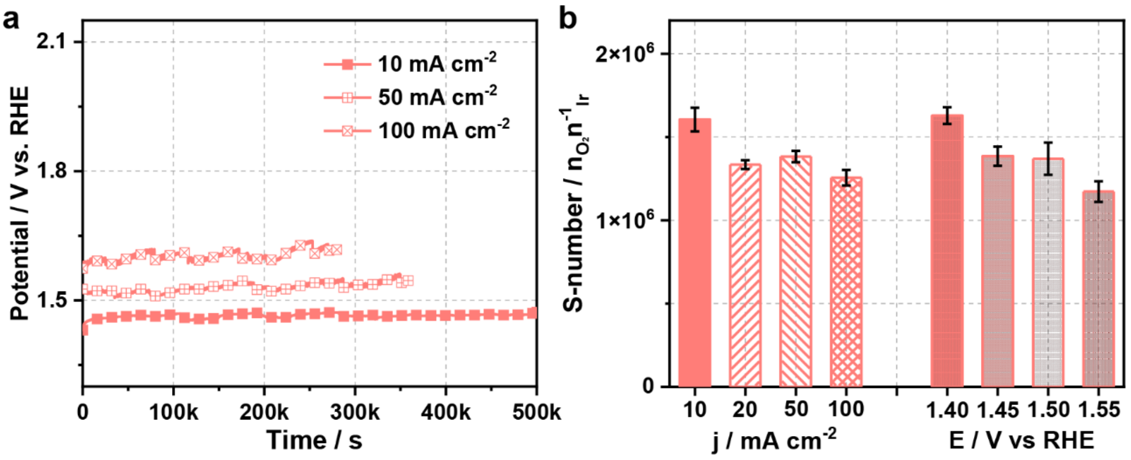

Supplementary Figure 30. Stability tests for IrO_2NR. (**a**) Chronopotentiometric curves of IrO_2NR at a constant current density of 10, 50 and 100 mA cm^{-2}; and (**b**) S-numbers of IrO_2NR at different current densities and different applied potentials (Error bars represent standard deviation, n = 3 independent replicates).

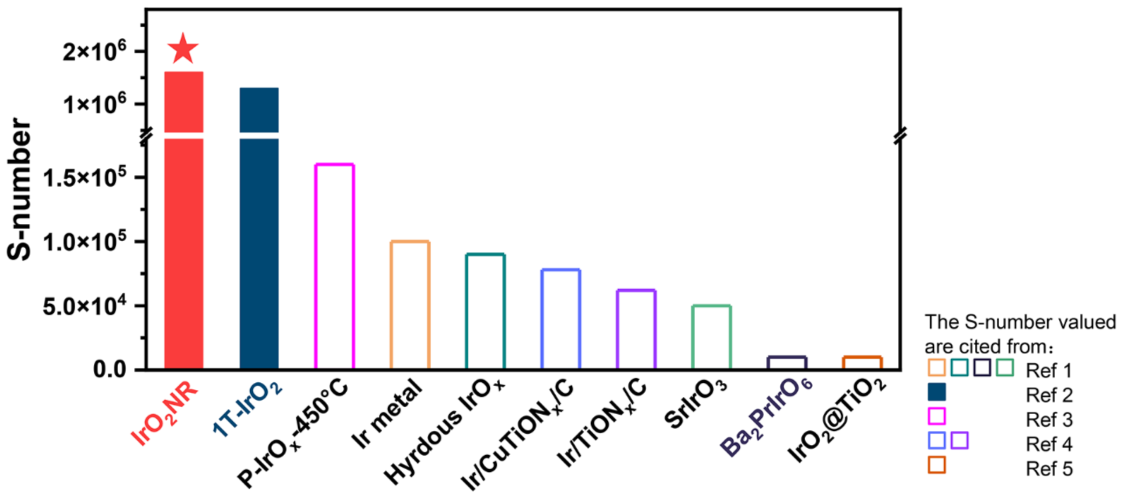

Supplementary Figure 31. Comparison of S-number values. S-number values of IrO_2NR compared with other reported iridium-based OER catalysts[1-5].

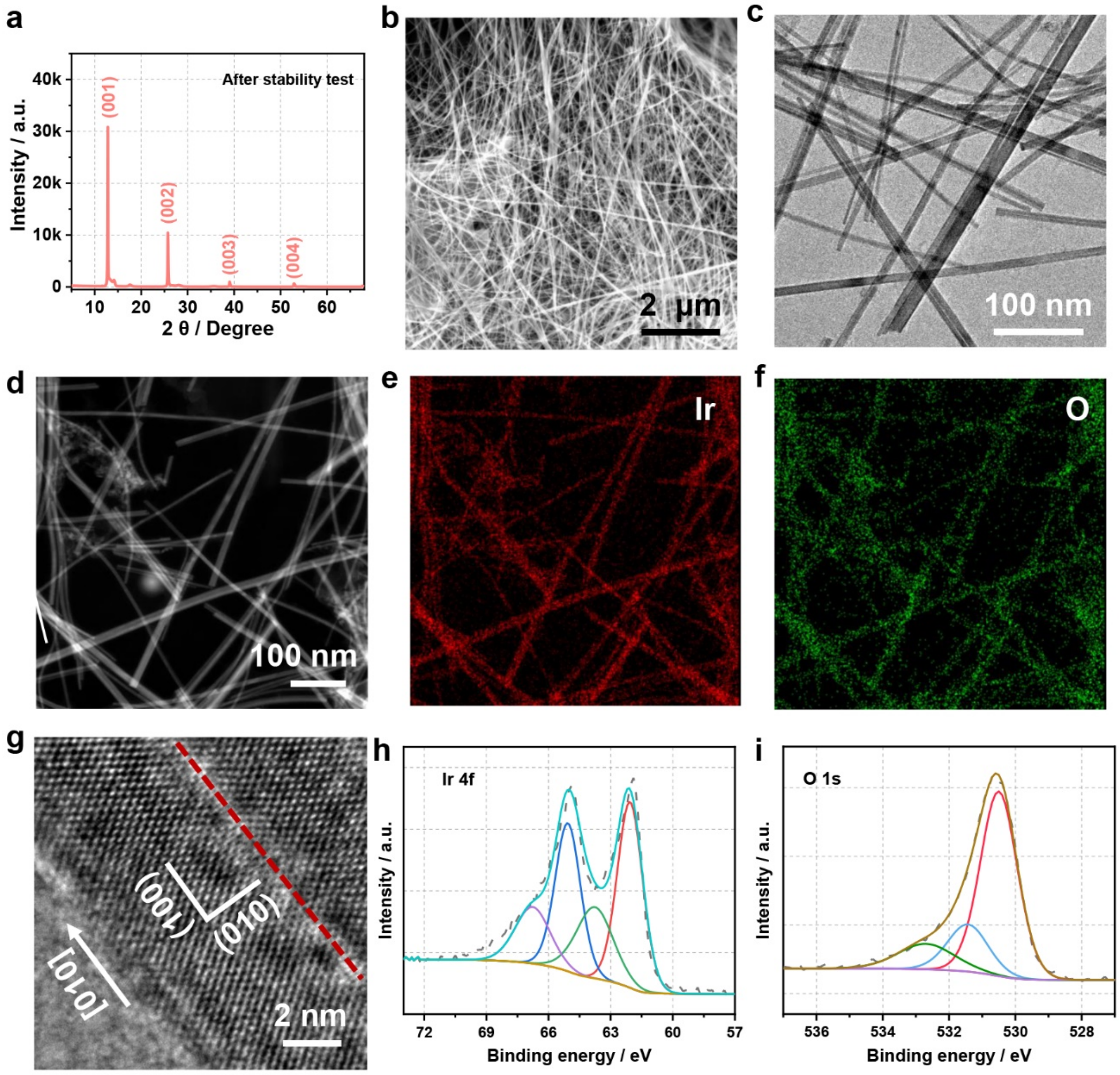

Supplementary Figure 32. Structure of IrO_2NR after long stability test with the constant current density of 10 mA cm^{-2} in 0.5 M H_2SO_4 electrolyte. (**a**) XRD pattern showing the maintained layered structure of IrO_2NR. (**b**) SEM image, (**c**) TEM image, (**d**) HAADF-STEM image and corresponding elemental EDS mapping image showing the distribution of (**e**) Ir and (**f**) O elements. The IrO_2NR are uniformly dispersed without aggregation or morphology change after undergoing the long-term OER. (**g**) HRTEM image showing clearly the regular atomic arrangement. The XPS spectra of (**h**) Ir 4*f* peak and (**i**) O 1*s* peak. The valence state of Ir keeps almost constant with the original IrO_2NR.

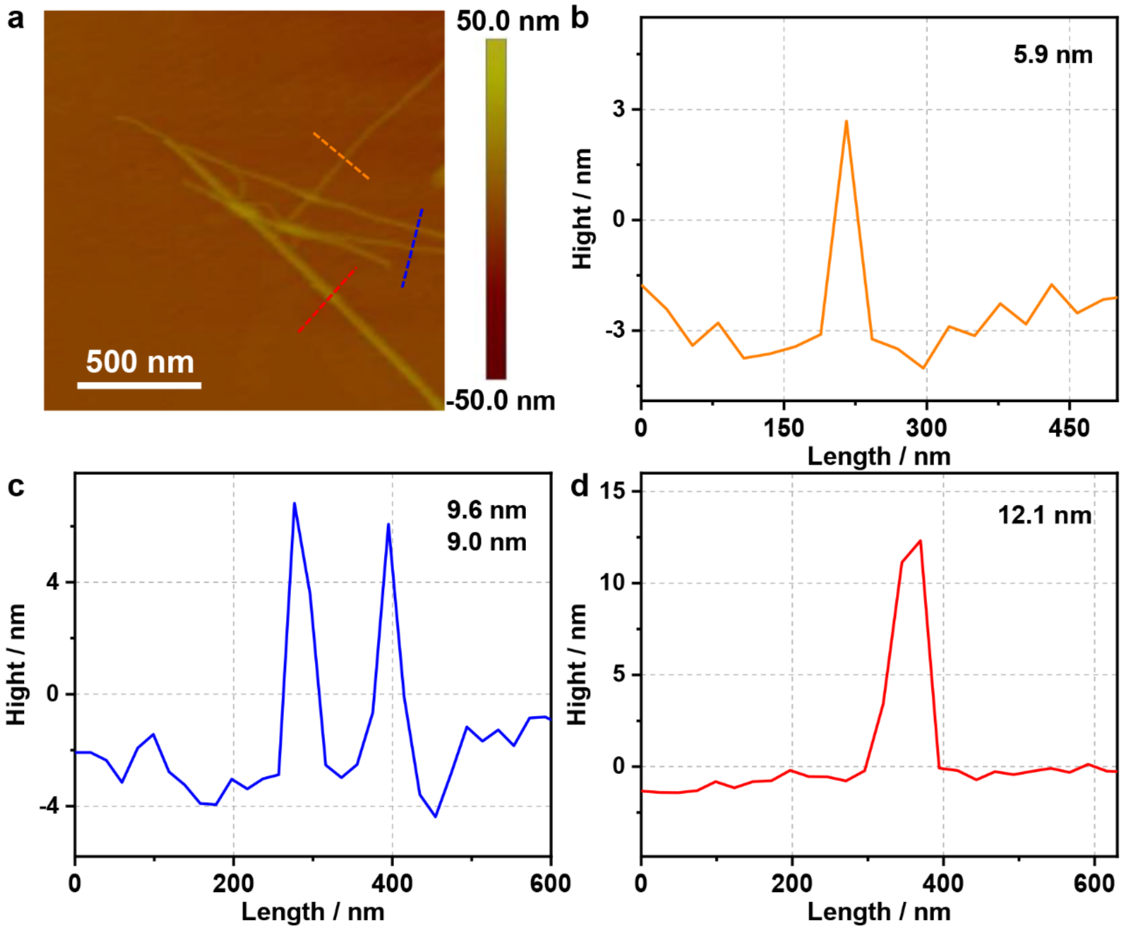

Supplementary Figure 33. Thickness of IrO_2NR after stability test. (**a**) AFM image and (**b-d**) the corresponding height profiles showing the thickness of the IrO_2NR.

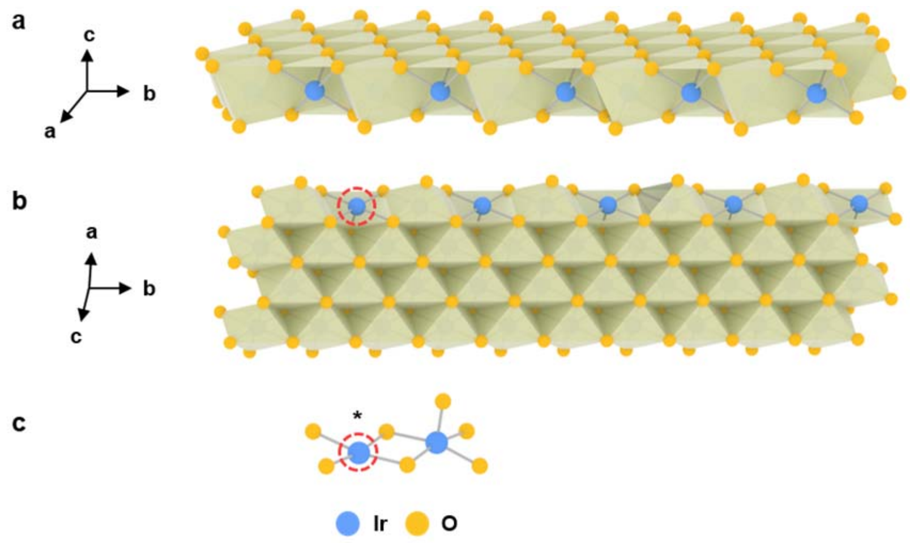

Supplementary Figure 34. Structure of IrO_2NR. (**a**) and (**b**) Crystal structures of the IrO_2NR with an exposed edge for the (001) plane during theoretical calculation with different viewing angles. (**c**) The ball-and-stick model extracted from (**b**) to show the surface location of active Ir in Figure 4c.

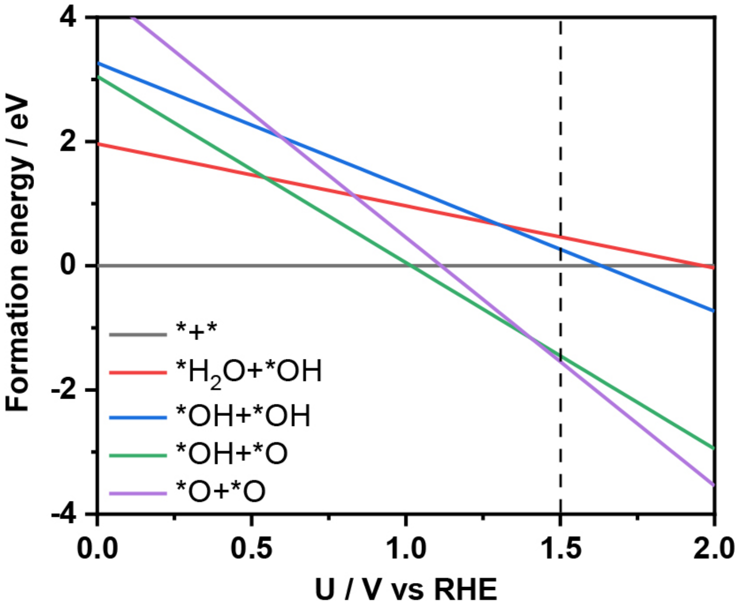

Supplementary Figure 35. Surface termination of the IrO_2NR under acidic conditions. Theoretical Pourbaix diagram of IrO_2NR (100) surface.

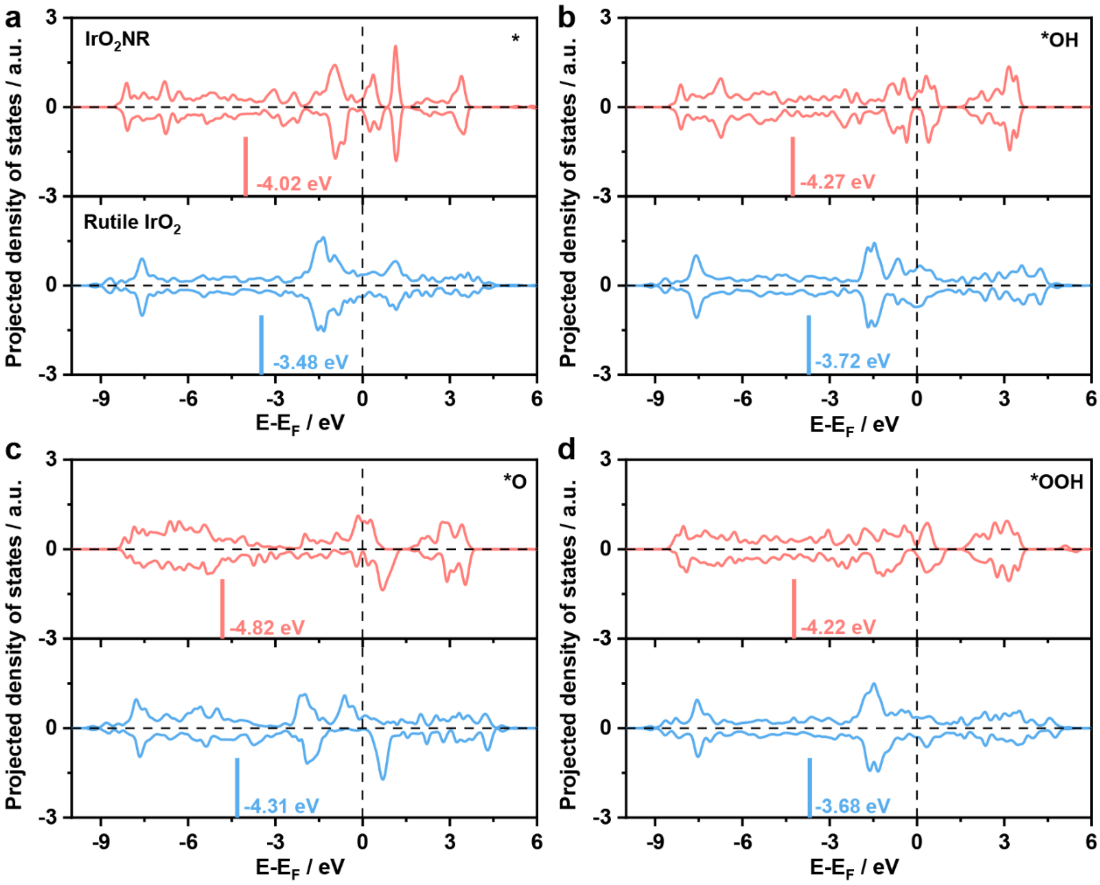

Supplementary Figure 36. The electronic properties of the Ir atoms. Comparison of *d*-orbital distribution of Ir atoms for the (**a**) *, (**b**) *OH, (**c**) *O and (**d**) *OOH in IrO_2NR (red curve) and Rutile IrO_2 (blue curve).

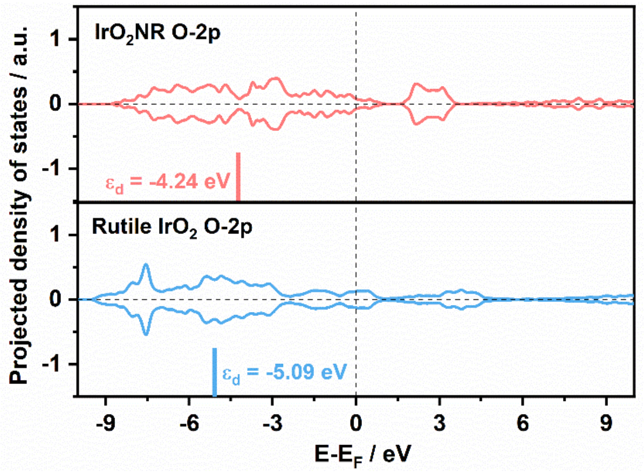

Supplementary Figure 37. The electronic properties of the O atoms. Comparison of the 2*p*-orbital distribution of the O atoms in the Rutile IrO_2 and IrO_2NR.

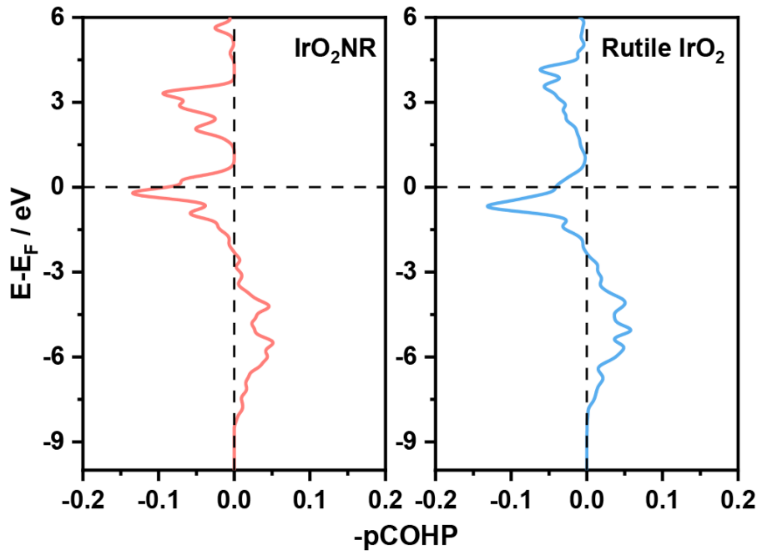

Supplementary Figure 38. Comparisons of projected crystal orbital Hamilton population. The Ir-O bonds in IrO_2NR (left) and the Ir-O bonds in Rutile IrO_2 (right).

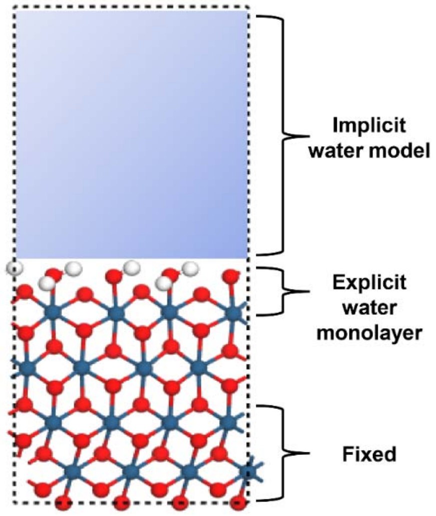

Supplementary Figure 39. Implicit solvation model. Schematic implicit solvation model for the calculation of Pourbaix diagram of IrO_2NR.

Supplementary Tables

Supplementary Table 1. Crystallographic information for IrO_2NR and C-IrO_2.

Material	Crystal system	Bravais lattice	Unit-cell dimension	Space group
**IrO_2NR**	**Monoclinic**	**C-centered**	**a = 4.43 ± 0.01 Å, b = 3.14 ± 0.02 Å, c = 6.95 ± 0.03 Å; $\alpha = \beta = \gamma$ = 90°**	**C2/m (12)**
C-IrO_2	Tetragonal	Primitive	$a = b$ = 4.498 Å, c = 3.154 Å; $\alpha = \beta = \gamma$ = 90°	$P4_2/mnm$ (136)
IrO_2NS	Trigonal	Primitive	$a = b$ = 3.11 Å, c = 6.91 Å; $\alpha = \beta$ = 90°, γ = 120°	P-3m1 (164)

Supplementary Table 2. The BET areas and ECSAs of IrO_2NR, C-IrO_2 and C-Ir/C.

Material	IrO_2NR	IrO_2NS	C-IrO_2	C-Ir/C
**BET area / m^2 g^{-1}**	47.3	22.0	11.8	397.6
**ECSA / m^2 g^{-1}**	31.3	21.6	15.5	16.3

Supplementary Table 3. OER activities of IrO_2NR, C-IrO_2 and C-Ir/C tested at 1.5 V vs RHE.

**Material**	**IrO_2NR**	**IrO_2NS**	**C-IrO_2**	**C-Ir/C**
**Overpotential @ 10 mA cm_{geo}^{-2} / mV**	205	235	303	322
**Tafel slope / mV dec^{-1}**	46.2	54.2	61.2	97.3
**Geometric activity / mA cm_{geo}^{-2}**	456.8	57.4	2.6	2.2
**Mass activity / mA mg_{Ir}^{-1}**	2354.5	295.9	13.4	48.6
**Specific activity / mA cm_{BET}^{-2}**	4.27	1.15	0.1	0.01
**Specific activity / mA cm_{ECSA}^{-2}**	6.45	1.17	0.07	0.3
**TOF_{BET} / s_{BET}^{-1}**	9.27	3.10	0.15	0.01
**TOF_{ECSA} / s_{ECSA}^{-1}**	14.01	3.07	0.12	0.3

Supplementary Table 4. Comparison of overpotential, Tafel slope and TOF of IrO_2NR with previously reported Ir-based catalysts.

Catalyst	Electrolyte	η@10 mA cm_{geo}^{-2} / mV	Tafel slope / mV dec^{-1}	Potential at 1 mA cm_{BET}^{-2}/ V	Mass activity / mA mg_{Ir}^{-1} @ Potential vs RHE	TOF/ s^{-1}@ Potential vs RHE	Ref
**IrO_2NR**	0.5 M H_2SO_4	**205**	**46.2**	**1.48**	**2354.5@1.50 V**	**9.27@1.50 V[a] 14.01@1.50 V[b]**	**This work**
3%IrO_2@BCNT		291	52	/	/	/	6
H-Ti@IrOx		277	29	1.58 V@ 0.04 mA cm_{ECSA}^{-2}	1500@1.58 V	/	7
IrRu@Te		220	35	/	590@1.50 V	/	8
$RuIrO_x$		233	42	/	/	/	9
$Rh_{22}Ir_{78}$/VXC		292	101	/	1174@1.53 V	5.095@1.53 V[b]	10
Li-IrO_x		300	39	1.55	1000@1.575 V	0.31@1.53 V[a]	11
IrO_x on IrCo		247	49	/	/	/	12
IrO_2/GCN		278	57	/	1280@1.60 V	0.175@1.60 V[d]	13
Ru@IrOx		282	69.1	/	644.8@1.56 V	/	14
Ir nanosheets		240	49	/	260@1.50 V	/	15
6H-$SrIrO_3$		248	/	1.55	75@1.525 V	/	16
Ir/GF		290	46	/	200@1.62 V	/	17
IrO_2/CNT		293	67	/	11.2@1.53 V	/	18
IrCoNi/CFP		303	54	/	750@1.53 V	0.26@1.53 V[a]	19
IrO_2-RuO_2@Ru		281	53.1	/	/	0.039@1.55 V[d]	20
IrO_x-Ir		290	~44	/	100@1.53 V	~1.1@1.53 V[b]	21
IrO_x/$SrIrO_3$		270	/	1.47	/	/	22
$IrNiO_x$		/	/	/	676@1.53 V	/	23
La_2LiIrO_6		/	/	/	~13@1.50 V	/	24
1T-IrO_2		235	49	/	152@1.50 V	2.0@1.50 V[b]	2
ultrathin iridium		328	45.4	/	209@1.53 V	/	25

nanosheets							
S-doped M-$SrIrO_3$ nanosheets		228	58.4	/	41.5@1.55 V	/	26
Ir nanosheets		254	72.5	/	1200@1.55 V	0.25@1.56 V[c]	27
Mesoporous IrOx nanosheets		~250	47	/	~1300@1.55 V	/	28
IrO_2 nanoneedles	1 M H_2SO_4	313	57	1.55 V@0.03 mA cm_{ECSA}^{-2}	51.6@1.55 V	/	29
Ir nanodendrites	0.05 M H_2SO_4	400	56	1.57	100@1.52V	/	30
3R-IrO_2		188	52	/	690.4@1.50 V	5.7@1.50 V[b]	31
$SrCo_{0.9}Ir_{0.1}O_{3-\delta}$		~290	/	1.56 V@10 mA cm_{BET}^{-2}	/	2.56@1.50 V[a]	32
IrNiCu double-layered nanoframe	0.1 M $HClO_4$	~303	48	/	120@1.50 V	/	33
$Y_2Ir_2O_7$		/	50	/	100@1.54 V	/	34
$H_{3.6}IrO_4 \cdot 3.7H_2O$		/	/	1.51	/	/	35
npIrx-NS		~300	~54	/	/	/	36
Amorphous Ir nanosheets		255	40	/	221.8 @1.53 V	0.16@1.53 V[c]	37

[a]based on the surface number sites calculated on BET.

[b]based on the moles of surface metal atoms on ECSA.

[c]based on the total number sites of mass.

[d]based on the moles of surface metal atoms in mol.

Supplementary Table 5. comparison of BET, ECSA and specific activity of IrO_2NR with previously reported Ir-based catalysts.

Catalyst	BET / m^2 g^{-1}	Specific activity based on BET / mA cm_{BET}^{-2}	ECSA / m^2 g^{-1}	Specific activity based on ECSA / mA cm_{ECSA}^{-2}	Ref
**IrO_2NR**	**47.3**	**1@1.48 V vs RHE 4.3@1.50 V vs RHE**	**31.3**	**1@1.47 V vs RHE 6.4@1.50 V vs RHE**	**This work**
H-Ti@IrOx	/	/	/	0.04@1.58 V vs RHE	7
Li-IrO_x	27	1@1.55 V vs RHE	/	~1@1.53 V vs RHE	11
6H-$SrIrO_3$	0.3	~0.03@1.48 V vs RHE	/	/	16
1T-IrO_2	21.9	~1.3@1.55 V vs RHE	15.4	~1.8@1.55 V vs RHE	2
IrO_2 nanoneedles	196.5	0.031@1.55 V vs RHE	205.1	0.03@1.55 V vs RHE	29
Ir nanodendrites	39.2	1@1.57 V vs RHE	/	/	30
3R-IrO_2	14.8	~4.2@1.5 V vs RHE	14.0	~4.21@1.5 V vs RHE	31
$SrCo_{0.9}Ir_{0.1}O_{3-\delta}$	0.175	~1@1.48 V vs RHE	/	/	32
$H_{3.6}IrO_4\cdot 3.7H_2O$	4.01	1@1.51 V vs RHE	/	/	35

Supplementary Table 6. ICP-MS results of IrO_2NR under different current densities and potentials for 3600 s.

	Current density / mA cm^{-2}				Potential / V vs. RHE			
	**10**	**20**	**50**	**100**	**1.40**	**1.45**	**1.50**	**1.55**
Concentration of Ir (ppb)	0.107	0.255	0.616	1.378	0.009	0.067	0.362	1.128
nO2 (μmol)	94.2	186.6	467.0	948.8	7.1	47.9	258.4	689.1
S-number	1605016	1334671	1382921	1255874	1628672	1385454	1369837	1172582

1 **Supplementary Table 7. ZPVEs and entropic correction at 300 K.**

	H_2O	H_2	*O	*OH	*OOH
ZPVE (eV)	0.60	0.30	0.07	0.37	0.46
ΔS (eV)	0.59	0.41	0.09	0.12	0.16

2

Supplementary Notes

Supplementary Note 1. The simulated coordinates for IrO_2NR.

```
01-sub
O Ir
   1.00000000000000
     15.0000000000000000    0.0000000000000000    0.0000000000000000
      0.0000000000000000    5.4324110000000001    0.0000000000000000
      0.0000000000000000    0.0000000000000000   25.9223649999999992
   O    Ir
    19     8
Selective dynamics
Direct
  0.4190650000000034  0.2579479999999990  0.0259640000000019   F   F   F
  0.4190650000000034  0.9238489999999970  0.1248099999999965   F   F   F
  0.4471198203822812  0.6545491573385520  0.2340191417309872   T   T   T
  0.4733495942004957  0.4099674867967978  0.3457988609085443   T   T   T
  0.4510552718603226  0.1375283621595616  0.4332698356881374   T   T   T
  0.4190650000000034  0.7579479999999990  0.0259640000000019   F   F   F
  0.4190650000000034  0.4238489999999970  0.1248099999999965   F   F   F
  0.4422174904000905  0.1574790054584634  0.2325569340693466   T   T   T
  0.4620263186325192  0.9051790564068785  0.3406259311725576   T   T   T
  0.5809349999999966  0.0830339999999978  0.0000000000000000   F   F   F
  0.5809349999999966  0.7489360000000005  0.0988460000000018   F   F   F
  0.5809349999999966  0.4148380000000031  0.1976930000000010   F   F   F
  0.5995550574035357  0.1514906889058718  0.3109581747931270   T   T   T
  0.6251726828718004  0.9164016834594557  0.4147991366877084   T   T   T
  0.5809349999999966  0.5830339999999978  0.0000000000000000   F   F   F
  0.5809349999999966  0.2489360000000005  0.0988460000000018   F   F   F
  0.5809349999999966  0.9148380000000031  0.1976920000000035   F   F   F
  0.5992910936771227  0.6625385755164241  0.3085092669953869   T   T   T
  0.6136816569837211  0.4032003522254856  0.4074041802867656   T   T   T
  0.5000000000000000  0.5034419999999997  0.0624049999999983   F   F   F
  0.5000000000000000  0.1693429999999978  0.1612510000000000   F   F   F
  0.5220876905389951  0.9165325830657767  0.2704034770555377   T   T   T
  0.5465663529252881  0.6799750677583399  0.3792005652002331   T   T   T
  0.5000000000000000  0.0034419999999997  0.0624049999999983   F   F   F
  0.5000000000000000  0.6693429999999978  0.1612510000000000   F   F   F
  0.5240494336193062  0.4007618178081752  0.2718000858633810   T   T   T
  0.5386840228307030  0.1397281512224765  0.3865812420588636   T   T   T

02-OH
H O Ir
   1.00000000000000
```

```
   15.0000000000000000    0.0000000000000000    0.0000000000000000
    0.0000000000000000    5.4324110000000001    0.0000000000000000
    0.0000000000000000    0.0000000000000000   25.9223649999999992
   H    O    Ir
     1    20     8
Selective dynamics
Direct
  0.4869046722266670  0.6796695669154855  0.4734471476813779   T   T   T
  0.4190650000000034  0.2579479999999990  0.0259640000000019   F   F   F
  0.4190650000000034  0.9238489999999970  0.1248099999999965   F   F   F
  0.4493551104986169  0.6538115026585298  0.2352068924827673   T   T   T
  0.4685265947716387  0.4032705463194092  0.3444880126933831   T   T   T
  0.4460404200917793  0.1281450585898191  0.4336860409467891   T   T   T
  0.4190650000000034  0.7579479999999990  0.0259640000000019   F   F   F
  0.4190650000000034  0.4238489999999970  0.1248099999999965   F   F   F
  0.4391142014764139  0.1564624534972288  0.2315223094660780   T   T   T
  0.4559209689777723  0.9089744956201233  0.3396461914434053   T   T   T
  0.5809349999999966  0.0830339999999978  0.0000000000000000   F   F   F
  0.5809349999999966  0.7489360000000005  0.0988460000000018   F   F   F
  0.5809349999999966  0.4148380000000031  0.1976930000000010   F   F   F
  0.5991559407180789  0.1494965021496423  0.3113624108423823   T   T   T
  0.6159069397987182  0.9099919555610447  0.4173677061722748   T   T   T
  0.5809349999999966  0.5830339999999978  0.0000000000000000   F   F   F
  0.5809349999999966  0.2489360000000005  0.0988460000000018   F   F   F
  0.5809349999999966  0.9148380000000031  0.1976920000000035   F   F   F
  0.5930621796413594  0.6606447617978193  0.3061233218646185   T   T   T
  0.6056735869717875  0.4017952874591937  0.4077403919680206   T   T   T
  0.4537724124361938  0.6764993969329485  0.4406407748655715   T   T   T
  0.5000000000000000  0.5034419999999997  0.0624049999999983   F   F   F
  0.5000000000000000  0.1693429999999978  0.1612510000000000   F   F   F
  0.5201963175305339  0.9235384113552986  0.2702674644800035   T   T   T
  0.5345665558677728  0.6780359439945117  0.3807995373832371   T   T   T
  0.5000000000000000  0.0034419999999997  0.0624049999999983   F   F   F
  0.5000000000000000  0.6693429999999978  0.1612510000000000   F   F   F
  0.5228683837382202  0.3909902872523834  0.2716706941679512   T   T   T
  0.5331267307575491  0.1336485933205033  0.3863388366432710   T   T   T

03-O
O Ir
   1.00000000000000
   15.0000000000000000    0.0000000000000000    0.0000000000000000
    0.0000000000000000    5.4324110000000001    0.0000000000000000
    0.0000000000000000    0.0000000000000000   25.9223649999999992
   O    Ir
```

```
    20     8
Selective dynamics
Direct
  0.4190650000000034  0.2579479999999990  0.0259640000000019   F   F   F
  0.4190650000000034  0.9238489999999970  0.1248099999999965   F   F   F
  0.4506213435184632  0.6568840874230705  0.2358826528955412   T   T   T
  0.4674781563964113  0.4017191746660626  0.3431075694857109   T   T   T
  0.4573671385236630  0.1513213230981630  0.4375853242497879   T   T   T
  0.4190650000000034  0.7579479999999990  0.0259640000000019   F   F   F
  0.4190650000000034  0.4238489999999970  0.1248099999999965   F   F   F
  0.4395338541121853  0.1561349346689531  0.2316806931647079   T   T   T
  0.4624684101476215  0.9142992000138565  0.3413876735687739   T   T   T
  0.4583247969381578  0.6599673983021526  0.4360430519211883   T   T   T
  0.5809349999999966  0.0830339999999978  0.0000000000000000   F   F   F
  0.5809349999999966  0.7489360000000005  0.0988460000000018   F   F   F
  0.5809349999999966  0.4148380000000031  0.1976930000000010   F   F   F
  0.6016265308471409  0.1554368935451549  0.3102450175213794   T   T   T
  0.6223036866206140  0.9052844807512053  0.4086612247496957   T   T   T
  0.5809349999999966  0.5830339999999978  0.0000000000000000   F   F   F
  0.5809349999999966  0.2489360000000005  0.0988460000000018   F   F   F
  0.5809349999999966  0.9148380000000031  0.1976920000000035   F   F   F
  0.5909188039156626  0.6596605627479961  0.3051418404574474   T   T   T
  0.6194287127516339  0.4086377767262041  0.4051088904614694   T   T   T
  0.5000000000000000  0.5034419999999997  0.0624049999999983   F   F   F
  0.5000000000000000  0.1693429999999978  0.1612510000000000   F   F   F
  0.5217444420812252  0.9276692841804395  0.2709750305103393   T   T   T
  0.5371201908335426  0.6632725168640359  0.3840989215630520   T   T   T
  0.5000000000000000  0.0034419999999997  0.0624049999999983   F   F   F
  0.5000000000000000  0.6693429999999978  0.1612510000000000   F   F   F
  0.5224513120508993  0.3900244917680420  0.2712175215135299   T   T   T
  0.5376182709382881  0.1512397974224304  0.3867738537579117   T   T   T

04-OOH
H O Ir
   1.00000000000000
     15.0000000000000000    0.0000000000000000    0.0000000000000000
      0.0000000000000000    5.4324110000000001    0.0000000000000000
      0.0000000000000000    0.0000000000000000   25.9223649999999992
   H    O    Ir
     1    21     8
Selective dynamics
Direct
  0.4720329614738420  0.6464561274848877  0.5173192365208771   T   T   T
  0.4190650000000034  0.2579479999999990  0.0259640000000019   F   F   F
```

0.4190650000000034	0.9238489999999970	0.1248099999999965	F	F	F
0.4480437220497948	0.6550524967693305	0.2344693105191512	T	T	T
0.4715672711257574	0.4091725904050069	0.3448848023231698	T	T	T
0.4494696992809179	0.1443702587225965	0.4328245565288801	T	T	T
0.4190650000000034	0.7579479999999990	0.0259640000000019	F	F	F
0.4190650000000034	0.4238489999999970	0.1248099999999965	F	F	F
0.4407871240435655	0.1582154797030442	0.2319743664960739	T	T	T
0.4602890615263362	0.9094940953218242	0.3402952235302220	T	T	T
0.5809349999999966	0.0830339999999978	0.0000000000000000	F	F	F
0.5809349999999966	0.7489360000000005	0.0988460000000018	F	F	F
0.5809349999999966	0.4148380000000031	0.1976930000000010	F	F	F
0.5997887931942437	0.1520144686175755	0.3108272481400659	T	T	T
0.6231902310863675	0.9174138219553138	0.4141971464961759	T	T	T
0.5809349999999966	0.5830339999999978	0.0000000000000000	F	F	F
0.5809349999999966	0.2489360000000005	0.0988460000000018	F	F	F
0.5809349999999966	0.9148380000000031	0.1976920000000035	F	F	F
0.5976084005880233	0.6642132219336677	0.3075249493112006	T	T	T
0.6135707822523041	0.4033430571898000	0.4065116086074406	T	T	T
0.5185462945748707	0.6465715389818154	0.4898291958197232	T	T	T
0.4678132550128926	0.6707265026463550	0.4454923420111937	T	T	T
0.5000000000000000	0.5034419999999997	0.0624049999999983	F	F	F
0.5000000000000000	0.1693429999999978	0.1612510000000000	F	F	F
0.5215581759085860	0.9199915717490112	0.2704639004679797	T	T	T
0.5410569529877362	0.6781303171646037	0.3811651948603809	T	T	T
0.5000000000000000	0.0034419999999997	0.0624049999999983	F	F	F
0.5000000000000000	0.6693429999999978	0.1612510000000000	F	F	F
0.5240942114161827	0.3988690320131885	0.2717791338671527	T	T	T
0.5375125698806702	0.1415826832235977	0.3860381793981448	T	T	T

Supplementary Note 2. The simulated coordinates for Rutile IrO_2.

```
01-sub
O Ir
   1.00000000000000
     6.3790139999999997     0.0000000000000000     0.0000000000000000
     0.0000000000000000     6.4275140000000004     0.0000000000000000
     0.0000000000000000     0.0000000000000000    27.1048849999999995
   O    Ir
    33    16
Selective dynamics
Direct
  0.0000000000000000  0.5000000000000000  0.1127090000000024   F   F   F
  0.0000000000000000  0.0000000000000000  0.2312760000000011   F   F   F
  0.9998445680351926  0.5010017369335630  0.3521987881139923   T   T   T
  0.9994965058634052  0.0016695960319556  0.4694566958742364   T   T   T
  0.0000000000000000  0.5000000000000000  0.0218169999999986   F   F   F
  0.0000000000000000  0.0000000000000000  0.1403839999999974   F   F   F
  0.9998819793757620  0.5008972018637352  0.2594521381489161   T   T   T
  0.9996259223036761  0.0019192165512308  0.3788791698653289   T   T   T
  0.2500000000000000  0.8083540000000013  0.0672629999999970   F   F   F
  0.2500000000000000  0.3083540000000013  0.1858300000000028   F   F   F
  0.2494157646963543  0.8090788817774327  0.3055969149996448   T   T   T
  0.2495907823033064  0.3106286536138581  0.4253387398246818   T   T   T
  0.2500000000000000  0.1916459999999987  0.0672629999999970   F   F   F
  0.2500000000000000  0.6916459999999987  0.1858300000000028   F   F   F
  0.2493828154747827  0.1929000641092782  0.3054636508096125   T   T   T
  0.2495815292875117  0.6930558945980061  0.4250517030320726   T   T   T
  0.5000000000000000  0.5000000000000000  0.1127090000000024   F   F   F
  0.5000000000000000  0.0000000000000000  0.2312760000000011   F   F   F
  0.4998198059594037  0.5007391366584167  0.3505882334525952   T   T   T
  0.4995544737545041  0.0014153080660952  0.4696174333714332   T   T   T
  0.5000000000000000  0.5000000000000000  0.0218169999999986   F   F   F
  0.5000000000000000  0.0000000000000000  0.1403839999999974   F   F   F
  0.4998843037734757  0.5008953976136499  0.2596797790019997   T   T   T
  0.4995913062981768  0.0018333004320948  0.3790363607518746   T   T   T
  0.7500000000000000  0.8083540000000013  0.0672629999999970   F   F   F
  0.7500000000000000  0.3083540000000013  0.1858300000000028   F   F   F
  0.7502442101018467  0.8091002149315418  0.3056004516223246   T   T   T
  0.7494529891548373  0.3106332557036335  0.4253109183090793   T   T   T
  0.7500000000000000  0.1916459999999987  0.0672629999999970   F   F   F
  0.7500000000000000  0.6916459999999987  0.1858300000000028   F   F   F
  0.7502852598433740  0.1929070926387134  0.3054594489419337   T   T   T
  0.7494504559824992  0.6930585580597578  0.4250282178903759   T   T   T
  0.9992729854811668  0.5034711991814431  0.4964397147839072   T   T   T
```

```
    0.2500000000000000   0.5000000000000000   0.0672629999999970   F   F   F
    0.2500000000000000   0.0000000000000000   0.1858300000000028   F   F   F
    0.2475069015541578   0.5009915241560096   0.3054876608785878   T   T   T
    0.2492901012064446   0.0017640358393325   0.4252172614315575   T   T   T
    0.0000000000000000   0.0000000000000000   0.0672629999999970   F   F   F
    0.0000000000000000   0.5000000000000000   0.1858300000000028   F   F   F
    0.9998254840168888   0.0009951528151384   0.3055600425645030   T   T   T
    0.9995095101943213   0.5019398334215505   0.4296801104279055   T   T   T
    0.7500000000000000   0.5000000000000000   0.0672629999999970   F   F   F
    0.7500000000000000   0.0000000000000000   0.1858300000000028   F   F   F
    0.7522189572992751   0.5009991211868521   0.3054923822539625   T   T   T
    0.7498078830423235   0.0017579660655349   0.4252075578576048   T   T   T
    0.5000000000000000   0.0000000000000000   0.0672629999999970   F   F   F
    0.5000000000000000   0.5000000000000000   0.1858300000000028   F   F   F
    0.4998190680808338   0.0009988354452719   0.3056387029642698   T   T   T
    0.4995109677516775   0.5018519584487785   0.4217974338177495   T   T   T

02-OH
H O Ir
   1.00000000000000
     6.3790139999999997    0.0000000000000000    0.0000000000000000
     0.0000000000000000    6.4275140000000004    0.0000000000000000
     0.0000000000000000    0.0000000000000000   27.1048849999999995
   H    O    Ir
     1   34   16
Selective dynamics
Direct
    0.6338218103958381   0.5067305354378833   0.5109118083149641   T   T   T
    0.0000000000000000   0.5000000000000000   0.1127090000000024   F   F   F
    0.0000000000000000   0.0000000000000000   0.2312760000000011   F   F   F
    0.0002115946427766   0.5006692981053429   0.3518085521966868   T   T   T
   -0.0006099061285491   0.0012056575053149   0.4693501256153819   T   T   T
    0.0000000000000000   0.5000000000000000   0.0218169999999986   F   F   F
    0.0000000000000000   0.0000000000000000   0.1403839999999974   F   F   F
   -0.0001616623193010   0.5005377364994646   0.2597714134179485   T   T   T
   -0.0001995536195784   0.0014122165233374   0.3789067445012633   T   T   T
    0.2500000000000000   0.8083540000000013   0.0672629999999970   F   F   F
    0.2500000000000000   0.3083540000000013   0.1858300000000028   F   F   F
    0.2498668038117612   0.8087345875183251   0.3053588169655004   T   T   T
    0.2491681853642929   0.3099691856292135   0.4247271754293133   T   T   T
    0.2500000000000000   0.1916459999999987   0.0672629999999970   F   F   F
    0.2500000000000000   0.6916459999999987   0.1858300000000028   F   F   F
    0.2498793038273987   0.1926416531991275   0.3052695071543272   T   T   T
    0.2492589770498049   0.6928085795571646   0.4244340201172746   T   T   T
```

```
0.5000000000000000 0.5000000000000000 0.1127090000000024 F F F
0.5000000000000000 0.0000000000000000 0.2312760000000011 F F F
0.4996246563607662 0.5007058506002751 0.3512755880197697 T T T
0.5000965351963679 0.0011627109843875 0.4692013539045547 T T T
0.5000000000000000 0.5000000000000000 0.0218169999999986 F F F
0.5000000000000000 0.0000000000000000 0.1403839999999974 F F F
0.5001122421505795 0.5006116887766602 0.2596781999178207 T T T
0.4995845123596138 0.0014303246404307 0.3789413676719597 T T T
0.7500000000000000 0.8083540000000013 0.0672629999999970 F F F
0.7500000000000000 0.3083540000000013 0.1858300000000028 F F F
0.7500473298671265 0.8087843372403861 0.3053729732162634 T T T
0.7503705996796457 0.3097062221571728 0.4240462945554899 T T T
0.7500000000000000 0.1916459999999987 0.0672629999999970 F F F
0.7500000000000000 0.6916459999999987 0.1858300000000028 F F F
0.7500577850612676 0.1926522401496229 0.3052822621686517 T T T
0.7504789256230621 0.6930821964265689 0.4238444840446888 T T T
0.9919481473566180 0.5024887867277223 0.4953921166567681 T T T
0.4916258834264045 0.5026283776001284 0.4972301991872556 T T T
0.2500000000000000 0.5000000000000000 0.0672629999999970 F F F
0.2500000000000000 0.0000000000000000 0.1858300000000028 F F F
0.2496574493718979 0.5006550766711961 0.3055484969244238 T T T
0.2493632091686847 0.0013633269078379 0.4252728188657499 T T T
0.0000000000000000 0.0000000000000000 0.0672629999999970 F F F
0.0000000000000000 0.5000000000000000 0.1858300000000028 F F F
-0.0000243257944562 0.0007067859798631 0.3055622792645232 T T T
0.0003751045719662 0.5014558878886503 0.4286777017602615 T T T
0.7500000000000000 0.5000000000000000 0.0672629999999970 F F F
0.7500000000000000 0.0000000000000000 0.1858300000000028 F F F
0.7503421554100849 0.5007302908895575 0.3056135304513852 T T T
0.7499413235432304 0.0013226072190987 0.4251436777818714 T T T
0.5000000000000000 0.0000000000000000 0.0672629999999970 F F F
0.5000000000000000 0.5000000000000000 0.1858300000000028 F F F
0.4999342224940794 0.0007045927376406 0.3055960218412249 T T T
0.5004319618172495 0.5013785617706408 0.4252213354183028 T T T

03-O
O Ir
1.00000000000000
6.3790139999999997 0.0000000000000000 0.0000000000000000
0.0000000000000000 6.4275140000000004 0.0000000000000000
0.0000000000000000 0.0000000000000000 27.1048849999999995
O Ir
34 16
Selective dynamics
```

```
Direct
  0.0000000000000000  0.5000000000000000  0.1127090000000024   F   F   F
  0.0000000000000000  0.0000000000000000  0.2312760000000011   F   F   F
  0.9998689644201714  0.5009002032862475  0.3519682429236804   T   T   T
  0.9993344891123284  0.0018118839891236  0.4692436650365079   T   T   T
  0.0000000000000000  0.5000000000000000  0.0218169999999986   F   F   F
  0.0000000000000000  0.0000000000000000  0.1403839999999974   F   F   F
  0.9999333076180618  0.5008703815970384  0.2598442601605263   T   T   T
  0.9995413867453676  0.0020125748078738  0.3788676535902920   T   T   T
  0.2500000000000000  0.8083540000000013  0.0672629999999970   F   F   F
  0.2500000000000000  0.3083540000000013  0.1858300000000028   F   F   F
  0.2499127295844903  0.8088026942427462  0.3054991807877735   T   T   T
  0.2494234004703997  0.3105887187729434  0.4243089340770135   T   T   T
  0.2500000000000000  0.1916459999999987  0.0672629999999970   F   F   F
  0.2500000000000000  0.6916459999999987  0.1858300000000028   F   F   F
  0.2499135989388178  0.1932366051173780  0.3053566572894766   T   T   T
  0.2494245778724460  0.6934107911073747  0.4239175605647564   T   T   T
  0.5000000000000000  0.5000000000000000  0.1127090000000024   F   F   F
  0.5000000000000000  0.0000000000000000  0.2312760000000011   F   F   F
  0.4998618233891925  0.5009105452674546  0.3519674233019505   T   T   T
  0.4993441147968616  0.0018272829415091  0.4692435260638592   T   T   T
  0.5000000000000000  0.5000000000000000  0.0218169999999986   F   F   F
  0.5000000000000000  0.0000000000000000  0.1403839999999974   F   F   F
  0.4999336059489501  0.5008698334069562  0.2598441626926167   T   T   T
  0.4995363575278933  0.0020289141258509  0.3788676927922908   T   T   T
  0.7500000000000000  0.8083540000000013  0.0672629999999970   F   F   F
  0.7500000000000000  0.3083540000000013  0.1858300000000028   F   F   F
  0.7499129015292626  0.8088032403638902  0.3054996207517020   T   T   T
  0.7494394270523926  0.3105891976385393  0.4243026078236614   T   T   T
  0.7500000000000000  0.1916459999999987  0.0672629999999970   F   F   F
  0.7500000000000000  0.6916459999999987  0.1858300000000028   F   F   F
  0.7499134914126016  0.1932366406207862  0.3053565385032874   T   T   T
  0.7494132118942283  0.6934129428582144  0.4239109108329677   T   T   T
  0.9993185444826654  0.5037874790445082  0.4953276145215443   T   T   T
  0.4993734011744317  0.5036879808826554  0.4953242154944987   T   T   T
  0.2500000000000000  0.5000000000000000  0.0672629999999970   F   F   F
  0.2500000000000000  0.0000000000000000  0.1858300000000028   F   F   F
  0.2499169030561023  0.5009770799872626  0.3057677369430566   T   T   T
  0.2494291759223654  0.0019432604995545  0.4251638409715845   T   T   T
  0.0000000000000000  0.0000000000000000  0.0672629999999970   F   F   F
  0.0000000000000000  0.5000000000000000  0.1858300000000028   F   F   F
  0.9998892883281378  0.0010193371458187  0.3056226939312163   T   T   T
  0.9994073756976700  0.5021101886134252  0.4286552763636916   T   T   T
  0.7500000000000000  0.5000000000000000  0.0672629999999970   F   F   F
```

```
   0.7500000000000000   0.0000000000000000   0.1858300000000028     F    F    F
   0.7499182810285098   0.5009796978011276   0.3057687879354140     T    T    T
   0.7494295141586830   0.0019447972812111   0.4251619733868104     T    T    T
   0.5000000000000000   0.0000000000000000   0.0672629999999970     F    F    F
   0.5000000000000000   0.5000000000000000   0.1858300000000028     F    F    F
   0.4998887467097261   0.0010207903689170   0.3056225398537408     T    T    T
   0.4994103929723537   0.5021007607696568   0.4286544999212406     T    T    T

04-OOH
H O Ir
   1.00000000000000
     6.3790139999999997    0.0000000000000000    0.0000000000000000
     0.0000000000000000    6.4275140000000004    0.0000000000000000
     0.0000000000000000    0.0000000000000000   27.1048849999999995
   H    O    Ir
     1    35    16
Selective dynamics
Direct
   0.5297212590431493   0.4177615828041949   0.5609517968202882     T    T    T
   0.0000000000000000   0.5000000000000000   0.1127090000000024     F    F    F
   0.0000000000000000   0.0000000000000000   0.2312760000000011     F    F    F
  -0.0003451184592728   0.4944432468660376   0.3519644386127907     T    T    T
   0.0001882990651607 -0.0054455271463742   0.4691720724593572     T    T    T
   0.0000000000000000   0.5000000000000000   0.0218169999999986     F    F    F
   0.0000000000000000   0.0000000000000000   0.1403839999999974     F    F    F
   0.0001276516495146   0.4987379466838284   0.2597312933866688     T    T    T
  -0.0007298965815942 -0.0027922360552167   0.3787117324938758     T    T    T
   0.2500000000000000   0.8083540000000013   0.0672629999999970     F    F    F
   0.2500000000000000   0.3083540000000013   0.1858300000000028     F    F    F
   0.2498096361876740   0.8055174447751586   0.3054163506269030     T    T    T
   0.2510967212646291   0.3038137292842731   0.4248298644130823     T    T    T
   0.2500000000000000   0.1916459999999987   0.0672629999999970     F    F    F
   0.2500000000000000   0.6916459999999987   0.1858300000000028     F    F    F
   0.2498905817199911   0.1899157653294576   0.3053085905288905     T    T    T
   0.2472976696229873   0.6880587838577156   0.4228715500751000     T    T    T
   0.5000000000000000   0.5000000000000000   0.1127090000000024     F    F    F
   0.5000000000000000   0.0000000000000000   0.2312760000000011     F    F    F
   0.5005286551200049   0.4945350070325660   0.3515087287865385     T    T    T
   0.4985998470488681 -0.0108192852744165   0.4689134234697506     T    T    T
   0.5000000000000000   0.5000000000000000   0.0218169999999986     F    F    F
   0.5000000000000000   0.0000000000000000   0.1403839999999974     F    F    F
   0.4999508058355370   0.4988785258575315   0.2597686749591206     T    T    T
   0.5000766116819096 -0.0033615787454754   0.3787958157552416     T    T    T
   0.7500000000000000   0.8083540000000013   0.0672629999999970     F    F    F
```

0.7500000000000000	0.3083540000000013	0.1858300000000028	F	F	F
0.7500462242857603	0.8049686590591850	0.3053428337911113	T	T	T
0.7505304900937947	0.3037502268207589	0.4262380358970056	T	T	T
0.7500000000000000	0.1916459999999987	0.0672629999999970	F	F	F
0.7500000000000000	0.6916459999999987	0.1858300000000028	F	F	F
0.7501131133095940	0.1893299063095829	0.3052318979352714	T	T	T
0.7494399131834137	0.6866586398525100	0.4238359169425996	T	T	T
0.0070337350979972	0.5026060251618678	0.4955031256750325	T	T	T
0.5658966608529270	0.3662788821623024	0.5277136118529102	T	T	T
0.4762856737701269	0.5268733806416662	0.4981488362517004	T	T	T
0.2500000000000000	0.5000000000000000	0.0672629999999970	F	F	F
0.2500000000000000	0.0000000000000000	0.1858300000000028	F	F	F
0.2495569599619835	0.4978797297798127	0.3056818313388325	T	T	T
0.2494960075735005	-0.0046518753176292	0.4247810136447266	T	T	T
0.0000000000000000	0.0000000000000000	0.0672629999999970	F	F	F
0.0000000000000000	0.5000000000000000	0.1858300000000028	F	F	F
-0.0000968684984893	-0.0024551339087700	0.3054452958349328	T	T	T
-0.0002363537065004	0.4958766678608142	0.4288260701893173	T	T	T
0.7500000000000000	0.5000000000000000	0.0672629999999970	F	F	F
0.7500000000000000	0.0000000000000000	0.1858300000000028	F	F	F
0.7505418782974969	0.4967459283923896	0.3056142407824793	T	T	T
0.7497601130295259	-0.0042913429674015	0.4251003239125429	T	T	T
0.5000000000000000	0.0000000000000000	0.0672629999999970	F	F	F
0.5000000000000000	0.5000000000000000	0.1858300000000028	F	F	F
0.4999895325035672	-0.0024098543549113	0.3055359256907156	T	T	T
0.4988734466362944	0.4958633544827046	0.4266112243991225	T	T	T

Supplementary Note 3. The code input file.

```
SYSTEM   =   Opt

ISYM      =   0
ISPIN     =   2

PREC      =   Normal
ALGO      =   FAST

ENCUT     =   520
EDIFF     =   1E-04
EDIFFG   =   -0.03

ISMEAR   =   0
SIGMA     =   0.05

LREAL     =   Auto
LWAVE     =   .FALSE.
LCHARG   =   .FALSE.

NSW       =   999
IBRION   =   2

GGA       =   RP
```

Supplementary References

1. Geiger, S., Kasian, O., Ledendecker, M., Pizzutilo, E., Mingers, A. M., Fu, W. T., Diaz-Morales, O., Li, Z. Z., Oellers, T., Fruchter, L., Ludwig, A., Mayrhofer, K. J. J., Koper, M. T. M. & Cherevko, S. The stability number as a metric for electrocatalyst stability benchmarking. *Nat. Catal.* **1**, 508-515 (2018).

2. Dang, Q., Lin, H. P., Fan, Z. L., Ma, L., Shao, Q., Ji, Y. J., Zheng, F. F., Geng, S. Z., Yang, S. Z., Kong, N. N., Zhu, W. X., Li, Y. Y., Liao, F., Huang, X. Q. & Shao, M. W. Iridium metallene oxide for acidic oxygen evolution catalysis. *Nat. Commun.* **12**, 6007 (2021).

3. da Silva, C. D. F., Claudel, F., Martin, V., Chattot, R., Abbou, S., Kumar, K., Jiménez-Morales, I., Cavaliere, S., Jones, D., Rozière, J., Solà-Hernandez, L., Beauger, C., Faustini, M., Peron, J., Gilles, B., Encinas, T., Piccolo, L., Barros de Lima, F. H., Dubau, L. & Maillard, F. Oxygen evolution reaction activity and stability benchmarks for supported and unsupported IrO_x electrocatalysts. *ACS Catal.* **11**, 4107-4116 (2021).

4. Lončar, A., Escalera-López, D., Ruiz-Zepeda, F., Hrnjić, A., Šala, M., Jovanovič, P., Bele, M., Cherevko, S. & Hodnik, N. Sacrificial Cu layer mediated the formation of an active and stable supported iridium oxygen evolution reaction electrocatalyst. *ACS Catal.* **11**, 12510-12519 (2021).

5. Pham, C. V., Bühler, M., Knöppel, J., Bierling, M., Seeberger, D., Escalera-López, D., Mayrhofer, K. J. J., Cherevko, S. & Thiele, S. IrO_2 coated TiO_2 core-shell microparticles advance performance of low loading proton exchange membrane water electrolyzers. *Appl. Catal. B: Environ.* **269**, 118762 (2020).

6. Liu, N., Duan, Z. Y., Zhang, Q. Q. & Guan, J. Q. Insights into active species of ultrafine iridium oxide nanoparticle electrocatalysts in hydrogen/oxygen evolution reactions. *Chem. Eng. J.* **419**, 129567 (2021).

7. Yu, Z. P., Xu, J. Y., Li, Y. F., Wei, B., Zhang, N., Li, Y., Bondarchuk, O., Miao, H, W., Araujo, A., Wang, Z, C., Faria, J. L., Liu, Y. Y. & Liu, L. F. Ultrafine oxygen-defective iridium oxide nanoclusters for efficient and durable water oxidation at high current densities in acidic media. *J. Mater. Chem. A* **8**, 24743-24751 (2020).

8. Xu, J. Y., Lian, Z., Wei, B., Li, Y., Bondarchuk, O., Zhang, N., Yu, Z. P., Araujo, A., Amorim, I., Wang, Z. C., Li, B. & Liu, L. F. Strong electronic coupling between ultrafine iridium-ruthenium

nanoclusters and conductive, acid-stable tellurium nanoparticle support for efficient and durable oxygen evolution in acidic and neutral media. *ACS Catal.* **10**, 3571-3579 (2020).

9. Zhuang, Z. W., Wang, Y., Xu, C. Q., Liu, S. J., Chen, C., Peng, Q., Zhuang, Z. B., Xiao, H., Pan, Y., Lu, S. Q. Yu, R., Cheong, W. C., Cao, X., Wu, K. L., Sun, K. A., Wang, Y., Wang, D. S., Li, J. & Li, Y. D. Three-dimensional open nano-netcage electrocatalysts for efficient pH-universal overall water splitting. *Nat. Commun.* **10**, 4875 (2019).

10. Guo, H. Y., Fang, Z. W., Li, H., Fernandez, D., Henkelman, G., Humphrey, S. M. & Yu, G. H. Rational design of rhodium-iridium alloy nanoparticles as highly active catalysts for acidic oxygen evolution. *ACS Nano* **13**, 13225-13234 (2019).

11. Gao, J. J., Xu, C. Q., Hung, S. F., Liu, W., Cai, W. Z., Zeng, Z. P., Jia, C. M., Chen, H. M., Xiao, H., Li, J., Huang, Y. Q. & Liu, B. Breaking long-range order in iridium oxide by alkali ion for efficient water oxidation. *J. Am. Chem. Soc.* **141**, 3014-3023 (2019).

12. Meng, G., Sun, W. M., Mon, A. A., Wu, X., Xia, L. Y., Han, A. J., Wang, Y., Zhuang, Z. B., Liu, J. F., Wang, D. S. & Li, Y. D. Strain regulation to optimize the acidic water oxidation performance of atomic-layer IrO_x. *Adv. Mater.* **31**, 1903616 (2019).

13. Chen, J. Y., Cui, P. X., Zhao, G. Q., Rui, K., Lao, M. M., Chen, Y. P., Zheng, X. S., Jiang, Y. Z., Pan, H. G., Dou, S. X. & Sun, W. P. Low-coordinate iridium oxide confined on graphitic carbon nitride for highly efficient oxygen evolution. *Angew. Chem. Int. Ed.* **58**, 12540-12544 (2019).

14. Shan, J. Q., Guo, C. X., Zhu, Y. H., Chen, S. M., Song, L., Jaroniec, M., Zheng, Y. & Qiao, S. Z. Charge-redistribution-enhanced nanocrystalline $Ru@IrO_x$ electrocatalysts for oxygen evolution in acidic media. *Chem* **5**, 445-459 (2019).

15. Jiang, B., Guo, Y. N., Kim. J., Whitten, A. E., Wook, K., Kani, K., Rowan, A. E., Henzie. J. & Yamauchi, Y. Mesoporous metallic iridium nanosheets. *J. Am. Chem. Soc.* **140**, 12434-12441 (2018).

16. Yang, L., Yu, G. T., Ai, X., Yan, W. S., Duan, H. L., Chen, W., Li, X. T., Wang, T., Zhang, C. H., Huang, X. R., Chen, J. S. & Zou, X. X. Efficient oxygen evolution electrocatalysis in acid by a perovskite with face-sharing IrO_6 octahedral dimers. *Nat. Commun.* **9**, 5236 (2018).

17. Zhang, J., Wang, G., Liao, Z, Q., Zhang, P. P., Wang, F. X., Zhuang, X. D., Zschech, E. & Feng, X. L. Iridium nanoparticles anchored on 3D graphite foam as a bifunctional electrocatalyst for excellent overall water splitting in acidic solution. *Nano Energy* **40**, 27-33 (2017).

18. Guan, J. Q., Li, D., Si, R., Miao, S., Zhang, F. X. & Li, C. Synthesis and demonstration of

subnanometric iridium oxide as highly efficient and robust water oxidation catalyst. *ACS Catal.* **7**, 5983-5986 (2017).

19. Feng, J. R., Lv, F., Zhuang, W. Y., Li, P. H., Wang, K., Yang, C., Wang, B., Yang, Y., Zhou, J. H. & Lin, F. Iridium-based multimetallic porous hollow nanocrystals for efficient overall-water-splitting catalysis. *Adv. Mater.* **29**, 1703798 (2017).

20. Li, G. Q., Li, S. T., Ge, J. J., Liu, C. P. & Xing, W. Discontinuously covered IrO_2-RuO_2@Ru electrocatalysts for the oxygen evolution reaction: how high activity and long-term durability can be simultaneously realized in the synergistic and hybrid nano-structure. *J. Mater. Chem. A* **5**, 17221-17229 (2017).

21. Lettenmeier, P., Wang, L., Golla-Schindler, U., Gazdzicki, P., Canas, N. A., Handl, M., Hiesgen, R., Hosseiny, S. S., Gago, A. S. & Friedrich, K. A. Nanosized IrO_x-Ir catalyst with relevant activity for anodes of proton exchange membrane electrolysis produced by a cost-effective procedure. *Angew. Chem. Int. Ed.* **55**, 742-746 (2016).

22. Seitz, L. C., Dickens, C. F., Nishio, K., Hikita, Y., Montoya, J., Doyle, A., Kirk, C., Vojvodic, A., Hwang, H. Y. & Norskov, J. k. A highly active and stable IrO_x/$SrIrO_3$ catalyst for the oxygen evolution reaction. *Science* **353**, 1011-1014 (2016).

23. Nong, H. N., Reier, T., Oh, H. S., Gliech, M., Paciok, P., Vu, T. H. T., Teschner, D., Heggen, M., Petkov, V., Schlogl, R., Jones, T. & Strasser, P. A unique oxygen ligand environment facilitates water oxidation in hole-doped $IrNiO_x$ core-shell electrocatalysts. *Nat. Catal.* **1**, 841-851 (2018).

24. Grimaud, A., Demortiere, A., Saubanere, M., Dachraoui, W., Duchamp, M., Doublet, M. L. & Tarascon, J. M. Activation of surface oxygen sites on an iridium-based model catalyst for the oxygen evolution reaction. *Nat. Energy* **2**, 16189 (2017).

25. Cheng, Z. F., Huang, B. L., Pi, Y. C., Li, L. G., Shao, Q. & Huang, X. Q. Partially hydroxylated ultrathin iridium nanosheets as efficient electrocatalysts for water splitting. *Natl. Sci. Rev.* **7**, 1340-1348 (2020).

26. You, M. S., Gui, L. Q., Ma, X., Wang, Z. B., Xu, Y., Zhang, J., Sun, J., He, B. B. & Zhao, L. Electronic tuning of $SrIrO_3$ perovskite nanosheets by sulfur incorporation to induce highly efficient and long-lasting oxygen evolution in acidic media. *Appl. Catal. B: Environ.* **298**, 120562 (2021).

27. Xie, Y. H., Long, X., Li, X. W., Chang, C. F., Qu, K. G. & Yang, Z. H. The template synthesis of ultrathin metallic Ir nanosheets as a robust electrocatalyst for acidic water splitting. *Chem. Commun.*

57, 8620-8623 (2021).
28. Jiang, B., Kim, J., Guo, Y. N., Wu, K. C. W., Alshehri, S. M., Ahamad, T., Alhokbany, N., Henzie, J. & Yamachi, Y. Efficient oxygen evolution on mesoporous IrOx nanosheets. *Catal. Sci. Technol.* **9**, 3697-3702 (2019).
29. Lim, J., Park, D., Jeon. S. S., Roh, C. W., Choi, J., Yoon, D., Park, M., Jung, H. & Lee, H. Ultrathin IrO_2 nanoneedles for electrochemical water oxidation. *Adv. Funct. Mater.* **28**, 1704796 (2018).
30. Oh, H. S., Nong, H. N., Reier, T., Gliech, M. & Strasser, P. Oxide-supported Ir nanodendrites with high activity and durability for the oxygen evolution reaction in acid PEM water electrolyzers. *Chem.Sci.* **6**, 3321-3328 (2015).
31. Fan, Z. L., Ji, Y. J., Shao, Q., Geng, S. Z., Zhu, W. X., Liu, Y., Liao, F., Hu, Z. W., Chang, Y. C., Pao, C. W., Li, Y, Y., Kang, Z. H. & Shao, M. W. Extraordinary acidic oxygen evolution on new phase 3R-iridium oxide. *Joule* **5**, 3221-3234 (2021).
32. Chen, Y. B., Li, H. Y., Wang, J. X., Du, Y. H., Xi, S. B., Sun, Y. M., Sherburne, M., Ager, J. M., Fisher, A. C. & Xu, Z. C. J. Exceptionally active iridium evolved from a pseudocubic perovskite for oxygen evolution in acid. *Nat. Commun.* **10**, 572 (2019).
33. Park, J., Sa, Y. J., Baik, H., Kwon, T., Joo, S. H. & Lee, K. Iridium-based multimetallic nanoframe@nanoframe structure: an efficient and robust electrocatalyst toward oxygen evolution reaction. *ACS Nano* **11**, 5500-5509 (2017).
34. Lebedev, D., Povia, M., Waltar, K., Abdala, P. M., Castelli, I, E., Fabbri, E., Blanco, M. V., Fedorov, A., Coperet, C., Marzari, N. & Schmidt, T. J. Highly active and stable iridium pyrochlores for oxygen evolution reaction. *Chem. Mater.* **29**, 5182-5191 (2017).
35. Zhang, R. H., Pearce, P. E., Pimenta, V., Cabana, J., Li, H. F., Dalla Corte, D. A., Abakumov, A. M., Rousse, G., Giaume, D., Deschamps, M. & Grimaud, A. First example of protonation of ruddlesden–popper Sr_2IrO_4: a route to enhanced water oxidation catalysts. *Chem. Mater.* **32**, 3499-3509 (2020).
36. Chatterjee, S., Peng, X., Intikhab, S., Zeng, G. S., Kariuki, N. N., Myers, D. J., Danilovic, N. & Snyder, J. Nanoporous iridium nanosheets for polymer electrolyte membrane electrolysis. *Adv. Energy Mater.* **11**, 2101438 (2021).
37. Wu, G., Zheng, X. S., Cui, P. X., Jiang, H. Y., Wang, X. Q., Qu, Y. T., Chen, W. X., Lin, Y., Li, H.,

Han, X., Hu, Y. M., Liu, P. G., Zhang, Q. H., Ge, J. J., Yao, Y. C., Sun, R. B., Wu, Y. E., Gu, L., Hong, X. & Li, Y. D. A general synthesis approach for amorphous noble metal nanosheets. *Nat. Commun.* **10**, 4855 (2019).
